# No safety net in the face of climate change: The case of pastoralists in Kunene Region, Namibia

**DOI:** 10.1371/journal.pone.0238982

**Published:** 2020-09-15

**Authors:** Emilia N. Inman, Richard J. Hobbs, Zivanai Tsvuura

**Affiliations:** 1 School of Biological Sciences, The University of Western Australia, Perth, Australia; 2 Multidisciplinary Research Centre, University of Namibia, Windhoek, Namibia; 3 Centre for Functional Biodiversity, School of Life Sciences, University of KwaZulu-Natal, Scottsville, Pietermaritzburg, South Africa; University of Leeds, UNITED KINGDOM

## Abstract

Over the past decade, pastoralists in Kunene Region, Namibia, have endured recurrent drought and flood events that have culminated in the loss of their primary form of livelihood–pastoralism. Most pastoralists are finding it difficult to sustain their livelihoods, and their communities have fallen into extreme poverty. Ecosystem-based Adaptation (EbA) approaches are increasingly acknowledged as having the potential to enhance the adaptive capacity of vulnerable communities. The first step is to develop an understanding of how affected communities live, their perceptions of and how they respond to climate change and the biophysical impacts of climate change in their communities. This study aims to collect this information in order to explore the use of EbA to help pastoralists adapt to climate change. We examined an isolated pastoral Himba community, to understand their perceptions, experiences and understanding of climate change and its related impacts on their livelihoods. A nested mixed-methods approach using structured interviews was employed to address the study objectives. Interview results revealed that pastoralists lack scientific knowledge of climate change, and they have no access to climate change information. Though pastoralists have coping and adaptation approaches at the community level (such as making gardens, fishing, etc.), these have become ineffective as climatic uncertainty and change persist. Furthermore, pastoralists no longer get benefits from the environment, such as food and fodder. Despite this, there are currently no biodiversity interventions at the community level to address the impacts of climate change. Pastoralists have indicated their adaptation needs, particularly the provision of water supply to grow food. This is an open avenue to explore EbA approaches, specifically ecological restoration, while still addressing the need of the pastoralists. There is an urgent need to develop new practical adaptation strategies, including restoration options that will strengthen their adaptive capacity.

## Introduction

In this era of accelerated climate change, there is an urgent need to understand how people are affected by and are dealing or coping with, climate change. This is especially true for the most vulnerable communities in the world. IPCC’s latest report [1] on the envisaged future impacts of climate change was, to many, a wake-up call that climate change is indeed here, and visible impacts are being experienced all over the world. Finding real solutions to help human communities adapt to these impacts is an urgent challenge [2].

Climate change impacts are projected to acutely affect arid and semi-arid rangelands, which cover approximately two-thirds of the African continent [3]. The majority of the population living in these areas, rely on natural resources for their livelihoods [3,4], making them vulnerable to climate change. Pastoralists and agro-pastoralists are one of the most affected groups all around the globe [5–8] as they have to respond to climatic variabilities relentlessly [9]. Pastoralists generally occupy less productive lands which are often poorly developed and suffer historical, political and economic marginalization [10]. The situation is exacerbated by deep rural poverty, limited government capacity, and exposure to synergistic challenges [4,5].

Climate change may severely impact livelihoods, food security, and health of pastoralists through its effects on livestock and livestock systems [11]. The impacts of projected climate change on livestock systems include changes in herbage growth, the composition of pastures and in herbage quality [5]. Climate-related risks may also lead to low milk yields and high livestock mortality in many pastoral communities leading to high poverty rates [8].

Climate change is a real problem to Namibia’s continued development process [12]. The country is already characterised by a harsh arid environment, low levels of precipitation, persistent droughts, and variable temperatures [12]. Other influences, such as population growth and severe inequality, will interact with changing rainfall patterns to intensify the situation [13]. Namibia’s vulnerability to climate change is particularly acute due to the existence of highly erratic climate and the high dependence by poor and rural populations on climate-sensitive livelihoods and climate-related natural resources [13]. The country has experienced natural disasters of various scales such as floods, droughts and wildfires [14]. Recently, the president of Namibia declared drought as a state of emergency—the third in six years after poor rainfall resulted in crop failure and scorched grazing fields [15]. Namibia’s climate is expected to become hotter and drier in the future, with more unpredictability in rainfall [12]. The available agricultural land can hardly support the people who depend on it, and the predicted reduction in rainfall will likely make these areas less suitable for agriculture. There is a recognised need for proactive adaptation and disaster management efforts to decrease the negative impacts of climate change and related disasters on ecosystems and people’s lives [16].

Knowledge of climatic perceptions and adaptations is a critical entry point for decision and policymakers to study how and where to improve the adaptive capacity of farmers [17]. Local knowledge, therefore, contributes to effective adaptation. The remarkable thing about building on the local knowledge is the fact that it promotes adaptive capacity that is suitable to farmers by endorsing and supporting locally established adaptations [15,16]. Farmers’ perception of climate change and variability is, therefore, a prerequisite for adaptation within rural farming communities [11,18,19]. Many authors have indeed acknowledged that perception is critical in adaptation [11,18–22]. For farmers to consider whether to adopt a specific adaptation measure, they must first perceive that climate change has undoubtedly taken place [18,21], and then respond to changes through adaptation [9]. Identifying the perceptions of farmers about climate change is, therefore, essential for understanding their potential adaptation approaches and supporting them in their initiatives [18,19]. This can then be merged with scientific knowledge to ensure effective adaptation policy planning. According to Schipper and others [23], to truly support the needs of local communities, expert advice and scientific information must be inclusive of local indigenous knowledge, and this information needs to be location-specific and more user friendly. People’s perceptions can reveal local concerns to shed light on the actual impacts of climate change and variability on their livelihoods [24].

Local ecological knowledge provides information on the status and use of biophysical components of the environment [25]. This is especially important when considering the use of Ecosystem-based Adaptation (EbA) strategies. We need to understand how communities live, how they respond to climate change and the biophysical impacts of climate change in their communities. Additionally, to inform EbA strategies, we need to understand how they cope and what benefits they get from their environment. EbA strategies should, therefore, take into consideration the multiple social, economic, and cultural aspects of local communities as part of an overall adaptation strategy [26]. This study aims to collect this information in order to explore the use of EbA to help pastoralists adapt to climate change. Several studies, e.g. [27–29], have highlighted the importance of EbA to help farmers adapt to climate change. Unlike other nature-based solutions for adapting to climate change, EbA entails a more inclusive and participatory approach [30].

Pramova and others [27] provided examples of EbA initiatives that farmers in some parts of Africa and around the world have successfully implemented. For instance, in West Africa (Niger, Mali and Burkina Faso) farmers have long been managing trees to reduce their sensitivity to climate variability through a continuous harvest of products. In Rajasthan, India, farmers maintain trees on croplands and sell fodder from these trees for a higher price during drought years. In the Padma floodplain in Bangladesh, farmers use mango-based multi-storey cropping systems to increase farmer resilience to climate-related and other shocks by providing various products all year round. EbA specifically targets those vulnerable to climate change, especially those dependent on ecosystems and their services for their livelihoods [16,31].

Important to consider when addressing climate change is the fact that; each community is unique, and thus, the focus needs to be location-specific and need-based, taking into consideration the unique circumstances of each vulnerable community. Moreover, just as the impacts of climate change vary according to location, the strategies employed to cope with these changes may also differ [32].

Though many studies have addressed the impacts of climate change on poor communities and explored their perceptions of climate change, few have focused on isolated tribes and communities, especially in Africa. Furthermore, limited studies have been done to inform Ecosystem-based Adaptation strategies and explore ecological restoration opportunities towards addressing the impacts of climate change. Previous studies on climate change adaptation and vulnerability in Namibia have focused mostly on agro-pastoralist farmers in north-central Namibia [33–36], particularly on Oshiwambo farmers. Most studies have focused on crop farming, despite pastoralists being one of the most vulnerable groups to climate change [10]. Three research questions guided this study: 1) What are the perceptions of climate change impacts, adaptation and vulnerability of pastoralists in Kunene, 2) What are the existing coping and adaptation activities used by pastoralists to adapt to climate change/variability and how effective are they in helping them adapt to climate change, 3) What are the impacts of climate change/variability on the vegetation cover, biodiversity and livestock as perceived by the pastoralists, and what biophysical interventions are implemented to address these impacts?

We examined an isolated pastoral Himba community, to understand their perceptions, experiences and understanding of climate change and its related impacts on their livelihoods. Specifically, we 1) described and assessed different forecasting methods used by the communities to predict the weather using local knowledge, 2) described the impacts of climate-related events (e.g. floods and droughts) and communities that are vulnerable to these events 3) determined coping and adaptation strategies used by pastoralists to adapt to the impacts of climate change/variability as well as barriers to adaptation, and finally, 4) determined the ecosystem benefits and biophysical impacts especially on plants and vegetation cover as perceived by the pastoralists, in order to propose EbA strategies of relevance to the Himba pastoral community. This study can help inform recommendations as to how pastoralists can diversify or adapt new strategies to reduce their vulnerability. It can also inform policymakers, particularly at the government level, on formulating strategies to improve livelihoods of pastoralists, mainly focusing on EbA.

### Background to the Himba pastoralists and Kunene Region

The Himba people are a pastoral ethnic group inhabiting the Kunene Region in Namibia, formerly called Kaokoland. Their main form of livelihood is based on keeping cattle, sheep and goats. It is difficult to tell precisely how old some of their customs and practices are because most of African history is mostly an oral record [37]. To the best of our knowledge, there is, therefore, no archaeological evidence to reconstruct the beginnings of pastoralism in Kunene or Namibia. The earliest record of cattle, goat and sheep herders were well established in southern Angola and presumably, in neighbouring Kunene, in the 16th century by Portuguese mariners [38]. “The Himba people are cherished as the last remnants of the `old Africa*'* [39] for many reasons such as holding onto their primitive lifestyle despite the progress of the 21^st^ century [40]”. Livestock is core to their cultural beliefs and practices, and provide a connection to their ancestors through sacrifice [38]. The Himba were one of the wealthiest cattle-herding groups in Africa [38]. This is before the stock losses in the devastating drought of the early 1980s, and several subsequent droughts, though in many cases they managed to rebuild their herds [38].

Harring (2001) described the Himba people as:

“*A distinct people because they are visually conspicuous; their bodies-tall*, *mostly naked*, *bejewelled*, *covered hair to foot with a red ochre and butter paste*. *These are immediately recognisable*. *Their land is an isolated*, *mountainous high desert*, *straddling the border between Namibia and Angola*. *It is inland on an escarpment above the Atlantic Ocean*, *a "wilderness" inhabited by free-roaming desert elephants and other exotic game species; a corner of the "real" Africa*, *relatively isolated*, *but accessible by anyone with a credit card*. *The Himba's visual distinctiveness is deliberate*. *Over the past hundred years the nearby peoples*, *like most Africans*, *have largely adjusted their dress in response to missionary and colonial demands*. *The Himba did not*, *remaining dressed in leather aprons*, *naked above the waist*. *This is not*, *entirely*, *an indication of their "isolation"; instead*, *it reflects a prosperous pastoral people with a strong will to continue their lives*”.

However, a once-prosperous tribe has been reduced to a people dependent on government drought relief, their land is no longer able to sustain them, and most of them have lost their livestock due to reoccurring droughts for the past decade. Furthermore, history has not been exactly kind to them either. According to Harring [41] for many decades, the cattle wealth of the Himba kept them prosperous, many of them refusing to work for the colonisers as they lived contented lives. They benefited greatly from cattle trade with other tribes and at the border of Angola. This allowed them to grow strong as a tribe. In 1921, however, the then colony (South Africa) closed the border between their land (Kunene) and other tribal lands, as well as enforcing control at the Angolan border in order stop the trade. This was devastating to the Himba because most of their traditional trade routes were therefore no longer available. South Africa planned to segregate Kunene and destroy the Himba pastoral economy in order to coerce them into signing migrant labour contracts. However, the Himba cattle economy continued to grow because they kept cattle for social and political reasons as well. Their cattle economy was so parsimonious and efficient that it expanded, even when their trade outlets were abridged. South Africa then prohibited labour recruitment among the Himbas and in the 1980s built three army bases in their region, and the Himba were forced to move to fortified villages surrounded by barbed wires, under army watchtowers. Still, they tended their herds, although these were not prosperous times [41].

Though their traditional practices have contributed to the current situation of degradation, much of it has been shaped profoundly by a century of colonialism and climatic variability. For example, boundaries restricted their spatial mobility, a prohibition on livestock trading forced them into subsistence herding, and the forced internal relocation of large numbers of people led to environmental degradation [38]. The first main western technological intervention in Kunene started in the 1960s with the creation of artificial water points which resulted in severe land degradation [38]. Although this was a good initiative, it also interfered with the grazing systems that had been practised in the region for centuries. Before the creation of artificial water points, some areas were only used seasonally. As a result of the drilling programme, tens of thousands more cattle, goats and sheep were able to be supported during the dry season [38]. Today, Kunene is one of the most degraded regions in Namibia.

When times were good, the Himba had large herds, numbering up to five hundred per family, and grazing on common lands [41]. A 1972 survey reported that Kunene held 160,000 cattle, perhaps half-owned by the Himba [41]. Between 1979 and 1982, war and the worst drought on record devastated Kunene killing 80 to 90 per cent of the domestic stock in the early 1980s [38]. Still, they rebuilt their herds, but subsequent recurrent droughts during 1981, 1990, 1995, 1998, 2001, 2002, 2013, 2016 and 2018 [42,43] made it more and more challenging to retain their livestock. The drought of 2012/2013 was labelled as the worst drought in 30 years, and many pastoralists were left hopeless and unable to recover from the blow. The recent decade long drought decimated their livestock and led them into extreme poverty. Today political struggles of the 20th century have passed them by as they continue to practice their traditional way of life [44]; however, prosperous times are things of the past.

## Methodology

### Study area

The study was conducted in Epupa constituency (-17°00'13.97" S 13°14'53.70" E) in Kunene Region, Namibia. Namibia is situated in Southern Africa, neighbouring South Africa in the South, Botswana in the East and Angola in the North. Kunene region occupies the north-west corner of Namibia, and livestock production is one of the key sources of livelihood to many rural households. It has a population of 86 856 people, of which 17 696 live in Epupa Constituency [45]. Annual rainfall in Kunene is sporadic and increases from west to east from less than 50 to 415 mm [46]. The region has an arid climate like the rest of the country, and an abridged wet season, mainly extending from February to April and is characterised by dust storms particularly from August to October. The terrain is semi-arid and progressively becomes desert land towards the Skeleton Coast [47]. The most visible and readily felt impacts of climate change are perhaps the increasingly persistent dry spells and frequent droughts in this region. Farmers look forward to the rain every year, always on a lookout for available grazing land, but lack of rainfall has led to poor livestock condition and loss of animals [46]. Epupa Constituency has a literacy rate of 29% of the total population of the area, and 70% of the children from this constituency never attended school. Epupa is also the least developed constituency in the region, and about 77% of the population depends on farming as their primary source of income [47]. Epupa is categorised as the most impoverished constituency with 51% of the population classified as severely poor.

### The climatic situation of Kunene

**Fig 1:**
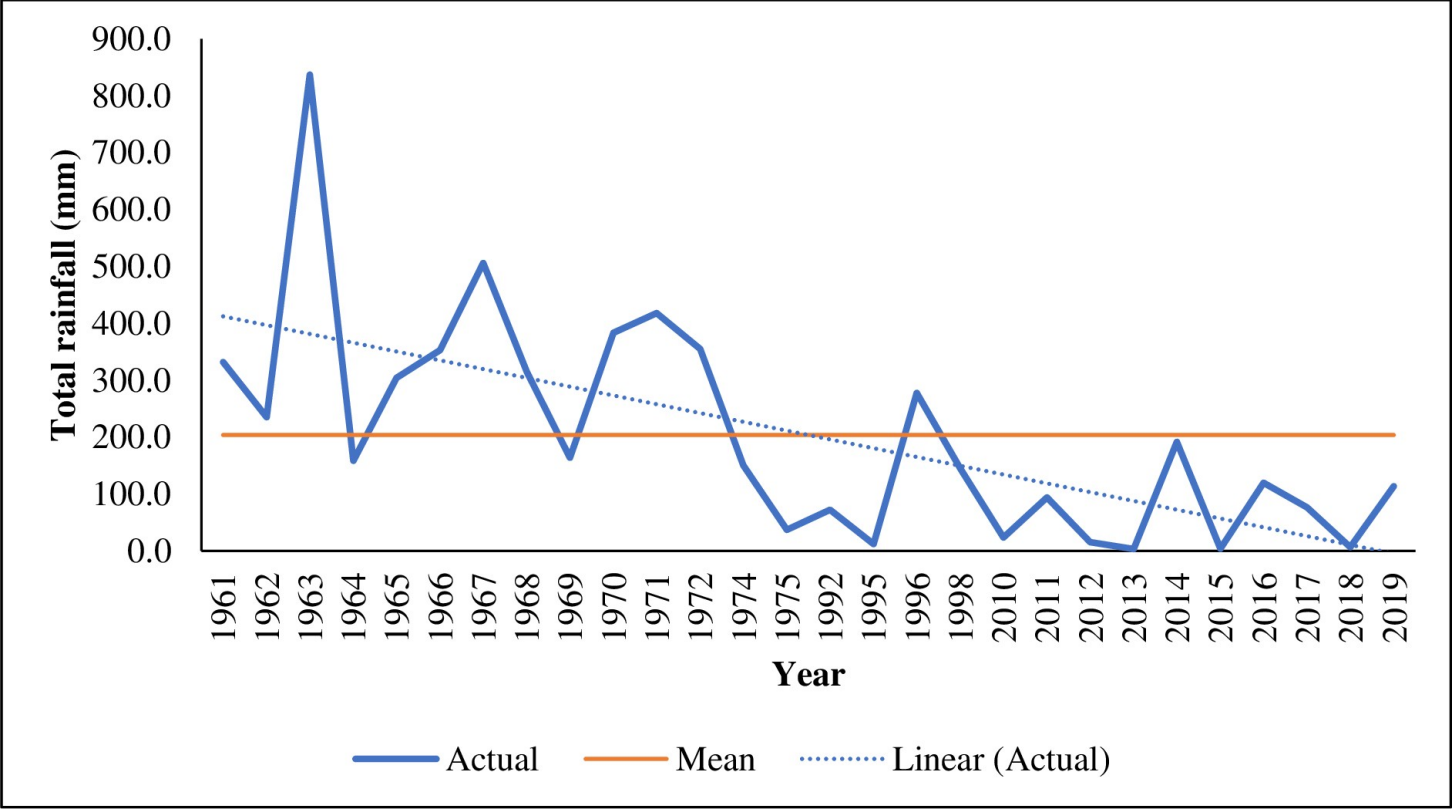
Total annual rainfall recorded at Opuwo town, Kunene Region from 1961–2019. Rainfall data was accessed by the corresponding author from the Namibia Meteorological Service data records in Windhoek on 25 February 2020. No rainfall data are available for the years 1976–1994 and 1999–2009.

In Kunene, there has only been one weather station (in Opuwo town) for the Namibian Meteorological Service since the 1960s. It was only recently that they installed another station in Okangwati, Kunene. The Opuwo station is about 110 km from the study site and has 59 years of records with some missing data. It is expected that extreme weather events such as floods and droughts will become more prevalent in Kunene [48], and this has already been happening. The onset of the rainy season will become more variable, and prolonged dry spells will also affect the development of functional grazing areas. Hunger is already pestilent among rural and poor populations in Kunene, exacerbated during prolonged drought conditions [48]. Fig 1 shows a drastic decrease in rainfall since 1962. Flood events are experienced even when it did not rain because, at the upstream of the Kunene River, there may be flood-inducing conditions that could bring floods to a drought-ravaged area downstream.

### Research design and sampling procedures

This study utilised a concurrent nested mixed-method research design. The term ‘concurrent’ shows that both qualitative and quantitative data are collected at the same time [49]. In concurrent nested studies, one of the methods dominates, and the other is embedded, or nested, in it [49]. In this case, the qualitative was the dominant method, while the quantitative data was embedded or nested within the larger qualitative study. We chose a qualitatively driven mixed-method approach because it allowed us to explore in-depth how climate change has impacted pastoralists’ livelihoods and hear their story. Qualitatively driven praxis stimulates a deep listening between the researcher and the researched, to get more in-depth and honest assertion of beliefs and values that surface through dialogue to uphold a more accurate description of views held [50]. Also, qualitative approaches tend to be open to new information and involve understanding people in their own terms, in their own social settings [50]. Qualitative dominant mixed methods research depend on a qualitative, constructivist critical view of the research process, while concurrently recognising that including quantitative data and approaches will likely add further benefit [51].

The qualitative component of the study consisted of open-ended questions which explored how pastoralists understand climate change, how it has affected them, how they cope or adapt to it, their perception of change and the environment as well as their vulnerability. The quantitative component was nested in the qualitative and consisted of closed-ended questions on demographic data, herd sizes, years of farming experience, frequency of drought and flood events, relief information and government’s involvement. The study being primarily qualitative, we used a combination of commonly used qualitative methods, namely in-depth interviews and structured interviews. We used the following sampling procedures–extreme cases, convenient procedures, snowballing and purposive sampling, commonly used in qualitative studies.

Due to dangerous road conditions, we conveniently selected villages that were accessible by a gravel road. For accessible villages, we randomly selected six villages, three of which were very close to Kunene River and three far from the river (over 25 km from the river). This was important to capture differences in adaptation opportunities. The distance was in terms of walking because people in the area lack vehicular transport. Kunene River is the main source of water for many people living in the area. They use water from the river for drinking, household use and for livestock.

The interviews were conducted between June and November 2018. All the interviews were carried out by the first author with her assistant as an interpreter who spoke the local language fluently, was born and grew up in the study area, and was therefore familiar with the local dynamics. The fact that one person conducted the interviews also meant bias was minimised in the data collection, although it also meant it was time-consuming. We are aware that our social identities are affected by our experiences with issues of class, race, ethnicity, and gender, and those social forces influence our world views, often defining our prejudice [52]. Although the lead author is a young black woman from a poor background similar to some Himba people, she has had the privilege of a good education, is well-travelled and has been exposed to people from all walks of life. Apart from this, she also belongs to a different tribe and cannot speak the Himba language. This perhaps could have made the Himba people perceive her as “different” or as an outsider. Some may have regarded her as someone in a position to help them, thus perhaps influencing them to make their case sound desperate. Growing up in similar circumstances as the Himba people, gave the lead author an advantage in that she was able to relate to what they were going through. However, this may have also tempted her to become too involved; thus, she maintained a neutral position as far as possible.

The research was conducted with the University of Western Australia Human Ethics Approval (RA/4/1/9296). It was made clear to the prospective respondents that the decision to take part in the interviews was voluntary. Individual informed consent to participate in the interviews was sought before each interview. A total of 16 key informant interviews, 16 in-depth interviews and 60 household interviews were conducted.

#### Description of methods used

Three methods were used to collect data, namely: (1) household interviews using a structured questionnaire, (2) key informants interviews using a structured questionnaire, and (3) in-depth interviews using structured questionnaires. The three methods are described below:

*Household interviews*. Household interviews included closed-ended and open-ended questions (to elicit quantitative and qualitative data, respectively). We collected information on socio-economic conditions, ownership and control over resources, land and livestock holding, utilization of tree species and ecosystem benefits, among others. Each interview was conducted with the household head, or if he or she was not available, his or her life partner was interviewed instead, if they had a partner.

For household interviews, we employed a snowball sampling procedure. This entails identifying respondents who are then used to refer researchers on to other respondents [53]. Using snowball strategies provide a means of accessing vulnerable and more impenetrable social groupings. This was especially important due to factors such as poor reception coverage, no radios and because the Himba people are very mobile and finding them at their homesteads especially men is difficult. The snowball procedure allowed us to recruit people with ease as news quickly spread, and more and more people wanted to express their concerns. Some respondents took us to their friend’s houses and even at water points, or in the fields and other places where we would not have been able to go. People were desperate to express themselves and their concerns, especially on the subject of drought. We usually always interviewed the headman first after obtaining permission, who then would invite some of his leaders or neighbours and news spread as more people were suggested and as the news spread, some families actually came to us, and we interviewed them under trees, at water points etc. We continued interviewing until we reached saturation. Theoretical saturation happens when adding study participants no longer produces new information, and, as a guide, is often achieved after approximately 15 interviews (+/- 10) [54]. We interviewed 30 homesteads in villages near the river, and by then, no new information was coming out at all. So we proceeded to the villages far from the river where we then used the same procedures for interviewing households and for uniformity also interviewed 30 households. The average interview length was one hour.

*Key informant interviews*. To collect detailed information on communities’ vulnerability, access to information, government intervention, etc., we focused upon a limited number of carefully selected community members (key informants). This comprised of community leaders and government or NGO workers such as teachers, police officers, etc., who had access to more information and outside help and had insights on people’s issues. Furthermore, they have also been working with the communities for more than five years and have access to information, and therefore able to give details of prevalent problems in the community. For selecting key informants, random sampling was entirely out of the question as only a few members fitted this criterion. So, we used purposive sampling according to the recommendations of some leaders as well as our own selection. We selected 16 key informants to meet the requirement for achieving saturation in qualitative interviews [54]. Scheduled appointments were carried out at respondent’s houses, or offices.

*In-depth interviews*. To get even deeper information of people’s issues and concerns, we carried out in-depth interviews focusing on extreme cases in the community, especially those who have lost most or all their livestock and have fallen into extreme poverty or those who had nothing at all. We also intended to understand the extent of vulnerability in the area through in-depth interviews. Furthermore, in-depth interviews provided more details on deeper social factors in the communities and more information on poverty and its link or interaction with climate change. The in-depth interviews were purposively selected [52] according to the recommendation of the headmen and other leaders, targeting extreme cases of poverty in the villages. We selected 16 pastoralists who participated in the in-depth interviews to meet the requirement for obtaining saturation which is usually about 15 interviews [54].

### Data analysis

The quantitative data were analysed using SPSS V. 25 [55] and Microsoft Excel 2016. Descriptive statistics were run to give frequencies and percentages of households’ socio-economic characteristics. For qualitative data, thematic analysis was carried out using NVIVO software version 12 [56] to identify different themes that came out of the interviews. Data were prepared for analysis by transcribing all the interviews, reducing the data into themes through a process of coding and representing the data. In the first stage, the coding process was guided by the main research question of the study, and some codes or themes were identified from the interview questions. Themes relevant to the objectives were identified to explain, compare or describe different phenomena. Secondly, data-driven coding followed with a focus on finding patterns of meaning. By reading through all the transcripts, more nodes and sub-nodes were identified that explained the patterns of the research questions from the interviewees’ perspectives. Transcripts were coded according to interviewees’ responses to each question, and the most prominent themes emerging across the set of interviews were identified. The interview questions also guided the coding process, mainly if there was a pattern in the responses of the participants, and critical ideas related to the research questions were identified as themes. Important points mentioned multiple times were also clustered together into a node or theme. Texts were coded to reflect the most frequently cited factors in response to a specific question asked.

## Results

### Socio-economic characteristics of the respondents

About 67% of the households were headed by males, while females headed 33% of the households. Most of the respondents (70%) were within the ages of 40–88 years, and the majority dwell in clumped mud houses with an average family size of nine persons. More than 70% of the respondents have never attended school, and this reflects the literacy rate of only 29% in Epupa Constituency (Table 1). The majority of the respondents (50%) were married, 21% were single parents, 16% were widowed, and the rest were divorced (8%) or cohabitating (5%). Pastoralism is the primary source of livelihood, and most respondents (57%) get their income from livestock production. About 16% depend on gardening or crops, 10% solely depend on drought relief or government pension. Some of them (4%) do not have gardens or livestock, and depend on activities such as fishing, making baskets, selling firewood, selling alcohol or food, and 2% depend on small-scale mining. Livestock species kept by households were mostly goats, selected for their drought resistance, followed by cattle and sheep. The participants were mainly living in non-brick houses (97%), and their main source of drinking water was either a river, tributary or human-made spring or well.

**Table 1:** Summary of respondents (n = 92).

Item	Category	N	Percentage%
Education	None	66	72
	Primary education	13	14
	Secondary education	7	8
	Tertiary education	6	6
Household head	Female-headed	30	33
	Male headed	62	67
Marital status	Cohabitating	5	5
	Divorced	7	8
	Married	46	50
	Single	19	21
	Widow	15	16
Family size	Average of family size	9	
Age	Average age	51	
Livelihood	Cattle and goat farming	52	57
	Drought relief	3	3
	Gardening/crops	15	16
	Government pension	6	7
	Small scale mining	2	2
	Government employees	10	11
	Others (such as selling baskets, alcohol)	4	4

### Perceptions of climate change

**Fig 2:**
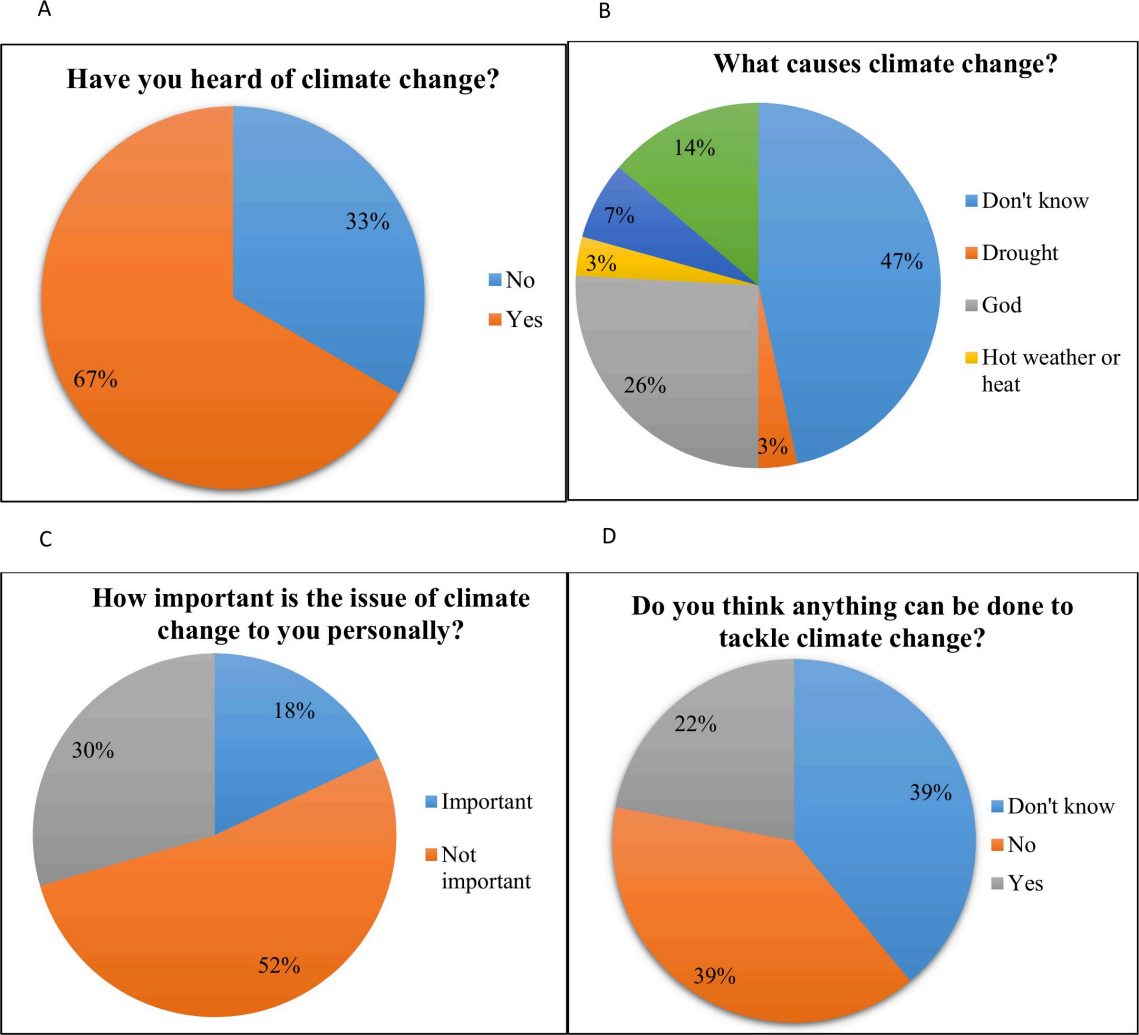
Perceived knowledge of causes, importance and beliefs of climate change among respondents (n = 60, household interviews).

About 67% of the respondents have heard of climate change (Fig 2A). The majority, including those who have heard of climate change, do not have an understanding of what climate change is and what causes it; thus, many expressed that it was not relevant to them. With regard to causes or reasons for climate change, many respondents (47%) did not know what causes climate change, and some (26%) expressed that it was caused by God (Fig 2B). More than half (52%) of the respondents noted that climate change was not really important to them (Fig 2C). The respondents were asked if they think anything can be done to tackle climate change. Many (39%) indicated that they do not know, and some (39%) expressed that there is nothing that can possibly be done to tackle climate change (Fig 2D).

When climate change was explained to them, the majority of the respondents expressed that they have perceived or experienced climate change or variability, especially delayed rainfall (98%), lack of rain (100%) and change in the temperatures (76%). Discussing climate change and explaining it to the community members seemed like a wake-up call, a new knowledge to them. Many expressed that though they have observed these changes, especially in the rainfall patterns, they did not understand why and what was causing all these changes (Table 2).

**Table 2:** Responses of some participants on their knowledge and beliefs of climate change.

Knowledge of climate change	Perceived climate change
*“I do not know anything about it*. *I have heard about it*, *but I do not have an understanding of what it is and what causes it*, *but we have seen these changes over many years” H49*	*“When I was young*, *though I did not pay much attention to the weather*, *I can see that things have changed now*. *We used to receive more rain*, *and it came on time*. *March to May is now the rainy season*, *but in the past*, *it used to come in October*, *and it rained until March or April” I10*
*“I do not really know anything about it” H1*	*“Some years*, *we receive rain and others not so much*, *several years can go by without any rain*, *sometimes even five years can pass without rain*. *We wonder why life is like this*, *but God is the one that gives all things” H19*
*“I just hear announcements on the radio about daily temperatures and wind direction etc*. *but we do not know anything about climate change and what causes it” H10*	*“When I was growing up*, *my parents told me and warned me that the world would change; the world will keep changing*. *The world is dynamic*, *and now I have observed they were right*. *Some years we get good rains*, *other years*, *not so good and I keep telling my children that things change*, *sometimes they become better and sometimes worse*, *but it will keep changing*, *and one needs to be ready for these changes” H2*
*“I hear about it on the radio*, *and I know it has become warmer this side of the river than it used to be years ago” H12*	*“Growing up*, *things were different*, *and we received enough rain*, *more than enough rain*, *now it is just drought” H6*
*“I know there are changes*, *and they are inevitable and up to God*, *and they will continue to happen*. *I know we may face any changes at any time*. *Suddenly there will be drought or flood; it will never be constant” H15*	*“I have observed these changes*. *So many changes and this brings me high blood pressure as I think about all the problems*, *and things are not getting better at all” H32*
*“I hear about it on the radio*, *but I have no idea what it is*. *How can someone know if something is important if they do not know it in the first place or have an understanding of it*, *but I guess if it is announced on the radio*, *it must be important” H56*	*“I just observe changes in different months*, *and sometimes the rain comes early*, *and other times*, *it is delayed*. *When drought occurs*, *it affects planting season and even plants in the wild are affected badly” H4*
*“Sometimes we hear from people discussing that the weather is changing and we talk about these changes with my friends” H5*	*The rain fluctuates and comes rarely*, *and for the past six years*, *we had no rain at all*. *The vegetation and all our crops just dry up*, *and we lose everything*, *crops*, *livestock etc*. *It is just hunger in this community*, *hunger for people and animals “H26*
*“I do not know anything*, *I just hear announcements on the radio*, *and I have observed these changes*, *and they make me really angry” H35*	* We have frequent droughts in this area; we are only living because of God “H33*
**Causes of climate change**	**Tackling climate change**
*“I do not know*, *and we see that these fluctuations have brought many bad things*, *but we do not know what causes these changes” H20*	*“I don’t think there is anything that anyone can do about climate change*, *not even the government*, *and that is because even in different countries where there are good hospitals*, *people are still dying every day*, *and human beings cannot do anything about this*, *in the same way*, *they can’t change climate change*. *It will not stop*, *it cannot be stopped because it is beyond us*, *just like death is inevitable” H10*
*“I do not know*, *and I do not give attention to it because it is something I cannot see “H10*	*“No*, *we are not God*, *we cannot do anything to change these things unless we can go to heaven and stop these changes” H3*
*“Drought causes climate change and God who is supreme” H11*	*“Only God can do something about climate change” H7*
*“I think they are brought by hot weathers” H12*	*“There is nothing we can do to avoid these changes” H21*
*“We do not know” H13*	*“Maybe we can pray to God*, *but it is truly hopeless” H37*
*“Climate change is caused by wind and rain” H14*	*“We need to pray harder and stop sinning*, *and all these bad things will stop*. *Things will change” H54*
*“I think God is responsible for these changes” H15*	*Maybe the government of perhaps international organisations could help to tackle these problems*. *If money is given*, *it can be used to help people make gardens and invest in education and in turn create jobs “H41*
*“The rain is the cause of climate change” H2*	*The elders in the community are the ones to decide on what could be done “H58*
*“These changes are brought by God because we do not pray enough” H21*	*We need to be educated and taught about these things so that we can do something about it “H2*

The majority of the pastoralists noted that they had no access to climate change (scientific) information. There was an indication that some were willing to do something about it if they had enough knowledge of what was expected of them. A 64-year-old pastoralist expressed that:

*“First of all*, *we need an understanding of what climate change is*, *what causes it*, *etc*. *We need to understand what brings rain*, *so that if there is something we are doing that is stopping the rain*, *then we can stop doing it*. *If there is something we can do to bring the rain*, *then we need to know so we can do these things*. *Without understanding*, *we cannot do anything at all” H2*

### Weather forecasting methods and indigenous knowledge

Our results revealed that there are no local campaigns or initiatives on climate information or early warning schemes in the study area. Many key informants (81%) and 100% of household interviewees indicated that there are no initiatives or campaigns to create awareness about climate change in the community. One of the pastoralists also expressed the need for awareness and early warning systems as follow:

*“People need awareness and to be taught about climate change*. *In this community*, *we are not taught anything; we do not even have cell phone coverage or radio to be informed*. *We are just lagging behind everyone*. *When the flood came*, *we were not informed*, *the flood came suddenly*, *but I heard on the other side people were warned through the radio about the oncoming flood” H21*.

Some respondents indicated that they use different forecasting methods to predict the rain such as bird movements, certain species of trees, wind patterns, phenology, presence and absence of certain animals, wind movements, moon and sun as indicators of whether they will have rain or not. Table 3 is a description of the indicators they use to predict the weather.

**Table 3:** Traditional forecasting used by pastoralists to predict the rain.

Type of change observed	Interpretation	Indicator Type
Many pods of mopane trees *(Colophospermum mopane)*	Drought	Biological
Less movement of birds	Drought	Biological
When we see flies with redheads and blue bodies	No rain	Biological
We observe the rainfall months if we do not get rain by December;	Drought	Meteorological
If the fruits start falling off before they ripen	Drought	Biological
If we get a westerly wind	No rain	Meteorological
If we receive easterly wind	There will be rain	Meteorological
We look at the Anna tree (*Faidherbia albida)* if we see no pods by July	There will be no rain	Biological
If the Marula trees *(Sclerocarya birrea*) produce fruits	Good rain	Biological
When we see sugar capsules secretions on mopane leaf	Good rain	Biological

Some elders use more complicated indicators such as looking at the guts of goats and observing the stars. These two statements are from two elderly pastoralists who use these methods:

*“We kill a goat*, *and then we look at the intestines and just by looking at the intestines*, *we can tell whether there will be drought or not*. *We dissect the goat and open the gut; in the intestines*, *there are glands that we look at that shows us that there will be drought or not*. *The glands are always there*, *but if there will be drought*, *they will be on another side or invisible*. *This to us is like weather instruments*, *and we use this accurately to tell us whether there will be drought or not” H18*

*“We also look at a group of stars and their position in the sky*, *we know by looking at them and where they are positioned in different times of the year*, *whether we will get rain or not” I8*

Nevertheless, the majority of the respondents (58%) stated that they are caught off guard because they can never tell when drought or flood will come. So they do not prepare for flood or drought, and even if they knew, they did not have the capacity to adequately prepare for either drought or flood.

### Perceptions of the impacts of climatic variability/change

**Fig 3:**
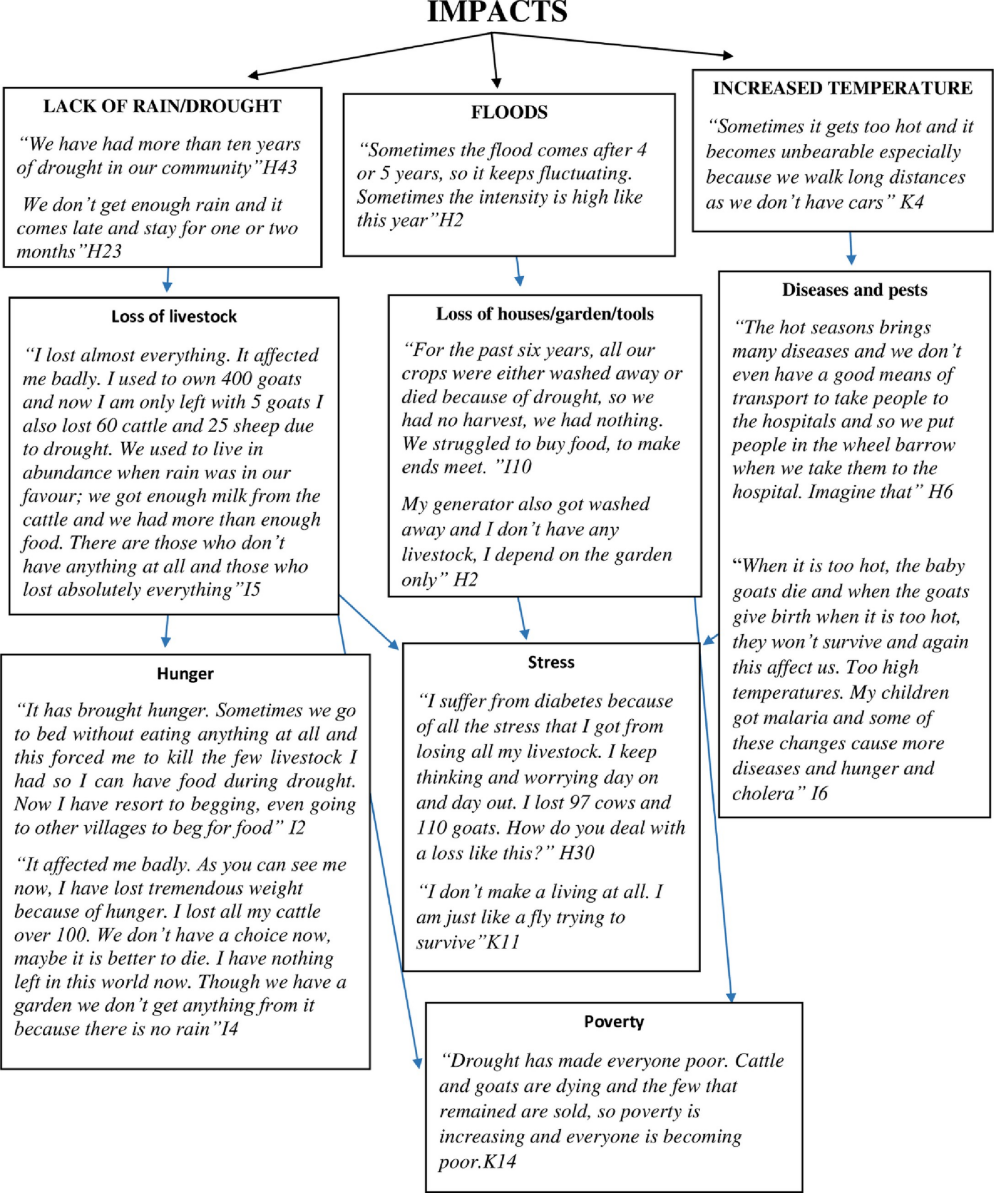
Impacts of climatic variability narrated by the pastoralists in Epupa, Kunene Region (household interviews).

Many respondents indicated that the climatic changes they have experienced have negatively impacted their lives; many complained of impacts such as drought, flood and high temperatures. Other impacts that were mentioned included diseases, hunger for both humans and animals and poverty (Fig 3).

#### Drought and floods

**Fig 4:**
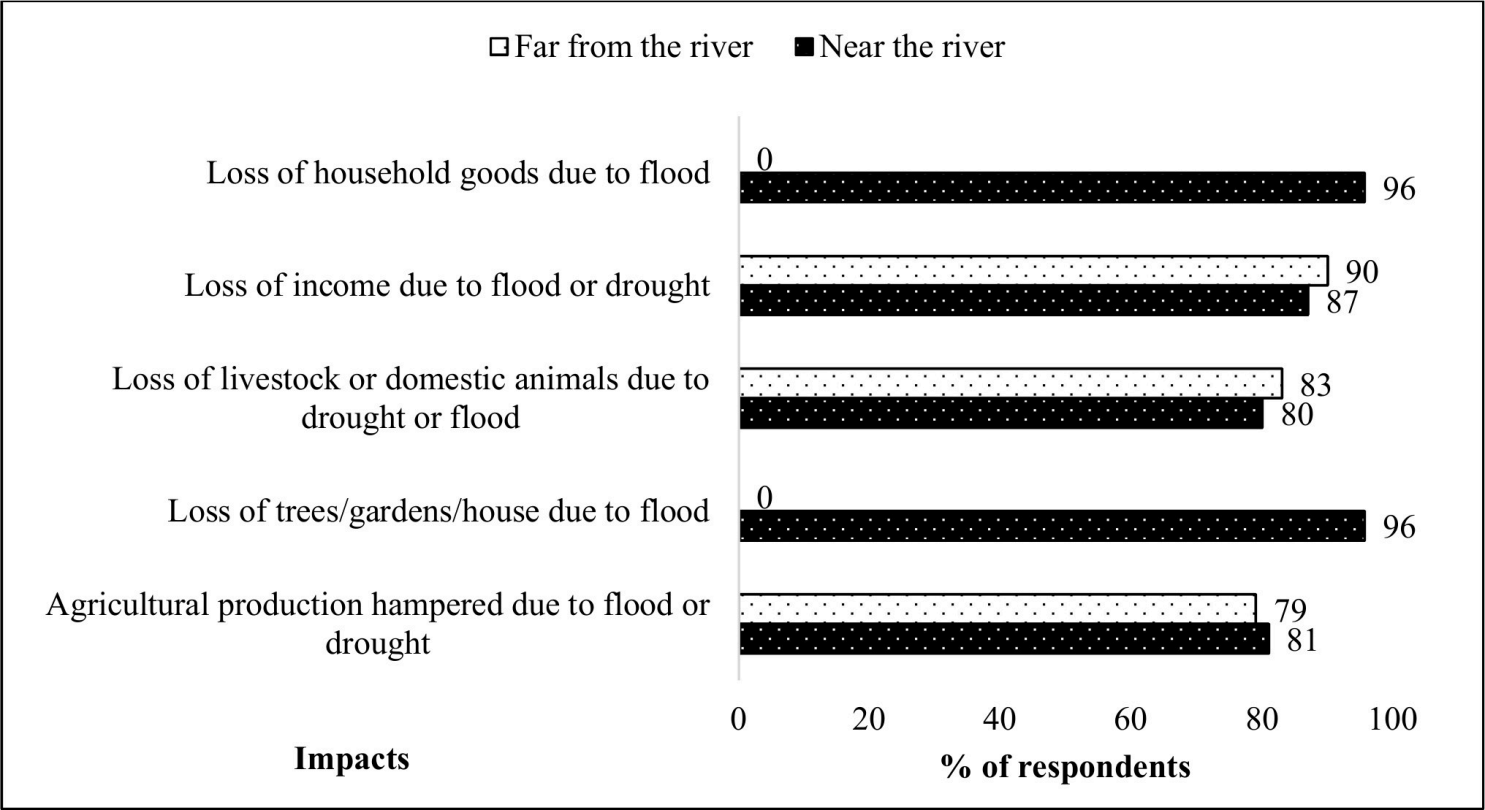
Perception of the effects of drought and flood (n = 60, household interviews).

Drought was a major theme that emerged from the study as an impact of climate change or variability. The majority of respondents (70–90%) were affected by the drought that has been reoccurring for the past decade, as have flood events. Those living near the river were more affected by floods, many losing their houses and household goods and their gardens washed away. Drought, however, affected everyone, many losing their livestock and experiencing reduced agricultural production (Fig 4).

Many pastoralists keep a record of their livestock and can remember how many livestock they have lost due to drought. The majority of pastoralists (84%) have lost several livestock. Based on in-depth interviews alone, 16 pastoralists’ estimates of the total livestock lost in the last five years was 2545 animals (Table 4).

**Table 4:** Number of livestock lost by individual respondents since 2015 (n = 16, in-depth interviews).

ID	2015–2018			Total livestock lost
	Number of cattle lost	Number of goats lost	Number of sheep lost	
I1	70	13	0	83
I2	7	0	0	7
I3	11	36	0	47
I4	100	125	0	225
I5	160	500	300	960
I6	5	0	0	5
I7	4	5	0	9
I8	7	1	0	8
I9	40	50	30	120
I10	15	11	0	26
I11	20	30	48	98
I12	100	0	0	100
I13	55	55	25	135
I14	153	290	0	443
I15	12	60	0	72
I16	97	110	0	207
				**2545**

The following three profiles of pastoralists illustrate to what extend drought has affected individual families in the area. The data presented is based on the interviews held with these pastoralists during in-depth interviews.

*Profile A*: *Pastoralist who keeps livestock for both subsistence and commercial purposes*. A 53 –year-old female pastoralist’s family has been farming for over 30 years. No one in the family has any formal education. They are a family of 11 people, owning 600 goats and 6 cattle, both for subsidence and commercial purposes. The drought in 2015 led to the loss of most of their cattle and some goats. Though they tried to save them by finding better pasture, it was to no avail. She reiterated that the drought situation has made them sad:

*“When I think about our lost cattle*, *it makes me sad*. *In 2016 we lost 50 cows*. *We tried so hard to save them*, *even taking them to places where we thought were better*, *but they died anyway*. *We were left with six cows only*. *A bag of 50 kg of flour in my house only lasts for a week; then I am required to purchase another one*. *I always need to have money to buy flour*, *and for this*, *I have to sell goats*. *I sold about five goats recently to make ends meet*. *I lost 11 goats in 2016*, *mostly because of livestock diseases and drought*. *I did not receive any help in terms of medicine etc*. *and no advice” I10*

Despite their loss, they are regarded as one of the most well-off families in the area; however, they may also be dragged into poverty if they continue to lose their livestock. They live in fear that recurrent drought may cause them even more harm.

*“During drought*, *I am concerned about the survival of our livestock because they are the most affected by drought*. *Drought affects our farm production and cattle are more affected by drought than goats*. *So my main concern is our cattle*. *I am afraid we may lose all we have*”.

*Profile B*: *Widower farming with livestock and a small garden*. A 68-year-old pastoralist has been farming for 51 years, with no formal education, and living with his four children. They have nine cows and a garden that they do not use because there is no rain and no means of irrigation. Asked about how the drought has affected them, he narrated that:

*“It affected me badly and my family*. *I lost 16 cattle in 2015*, *in 2016*, *I lost three and last year*, *and in 2017 I lost two cows*. *I lost goats and sheep too*, *about 30 goats and 48 sheep*. *When this happened*, *I lost my only source of income and means of livelihood*, *and now we live from hand to mouth*. *I wanted to take my life because there was nowhere to get food*. *I lost all my household goods due to this year’s flood*, *such as spades and hoes*, *including my garden*. *All gone*. *Even the rest of the communities are going through the same things” I11*

He said that he had been depressed and is fearing for the worst:

“*This has had bad effects on me*, *and it saddens my heart because I am worried where and how we will live in the future*. *Will I lose everything*? *Things that are supposed to keep us alive are gone or dead*, *no livestock and no crop production*. *When I see these things*, *I start thinking I am next in line*. *Even livestock diseases affect me badly because what affects my livestock directly affects me too*.

*Profile C*: *A headman with a secondary education*, *who has been farming for over 45 years*. A 78-year-old pastoralist and a headman has been farming since 1972. He is one of the few with secondary education and at some point worked for a veterinary office before he went into farming. He gave three accounts when he lost all his livestock due to drought in the area:

*“In 1993*, *I lost all my cattle*, *and in 1995 I started over*, *acquiring new cattle to sustain my family*.”

*“Drought affected my family badly*. *Since 2007 I lost many cattle*, *but since we had a lot*, *it was not so bad…*..*in 2015 I lost the remaining cattle*, *all 160 died*, *and I am left with 10 cattle*. *That was a hard blow*, *one that affected me badly*.

Apart from livestock production, he also invested in a garden a few years back, but it got washed away because it was too close to the river. During drought, they use buckets and fetch water from the river to grow what they can in the garden. Asked on how the drought has affected him, he narrated that:

*“Many years ago*, *I used to have many cattle*, *and now I only have ten*. *That is painful*, *and people who have lost livestock are severely affected*. *However*, *the worst are those who have nothing at all*. *Within 5 years*, *I lost about 160 cattle due to drought*. *I do not even want to talk about goats*. *I used to have so many goats; countless and sheep too*. *I lost 300 sheep and have lost more than 500 goats; now I only have 80 goats left and 15 sheep*. *All the goats I have now are new goats that I bought to start over*. *I keep adding here and there as years go by*. *I had to buy new goats because I lost everything*. *However*, *now I do not have money to buy more livestock and therefore have been left hopeless*.

Concerning the frequency of drought and flood events, many respondents indicated that they had experienced drought every year for the past six to ten years, while floods only come when it rains. The following statements are some views of pastoralists on the frequency of drought and floods:

*“Sometimes we go for 2 to 3 years without any flood event*, *and for the past six years*, *we had drought”* **H14**

*The flood fluctuates*, *sometimes we go for several years without experiencing any flood*, *but the drought is here every year”* **H16**

*“Drought is here every year*. *All we do is go and dig semi-precious stones to sell; otherwise*, *we will die of hunger*. *Flood only comes when it rains”* **H13**

Table 5 shows years of major drought events that some of the pastoralists could remember. Due to illiteracy, most were not able to recall the specific years and therefore referred to events such as when their children were born, and we had to work out which year by looking at their identification documents.

**Table 5:** Major droughts in the living memory of some pastoralists.

Period	Comments
1993	*1993 was a deadly year*, *terrible drought*, *I lost all my cattle and only started over in 1995*.
2007/2008	*In 2007*, *we had a severe drought for animals and people* ***H45***
	*We have been experiencing drought since 2007 to date* ***H34***
	*We have had more than ten years of drought since 2008*, ***H1***
2010/2011	*“In 2010 we had another severe drought*, *that killed many livestock and people too*. *This year the rain was very little”* ***H59***
	*2011 was a disastrous year for everyone here*, *and that is when we had the worst drought ever*. *Even people died*, *animals and humans died*, *and it was a tragedy*. *Even years after that we have never really recovered and drought continues-****H40***
2015	*“I lost my cattle again in 2015 because of a bad drought” I5*
2017/2018	*“In 2017 we moved our cattle to Angola for green pasture because we received very little rain” I15*
	*This year (2018)*, *there is drought again*, *no rain and our livestock are dyings*

### Local coping and adaptation strategies

**Fig 5:**
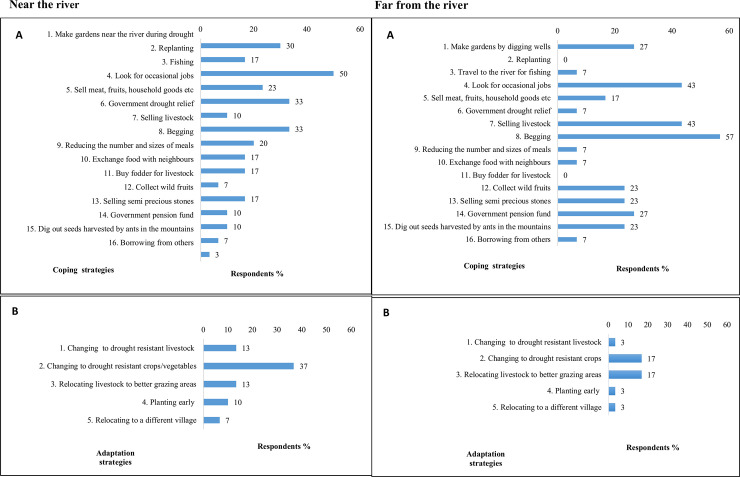
A) Coping and B) Adaptation strategies used by pastoralists living near and far from the river (n = 92).

Pastoralists in the study area employ a variety of coping (against short-term shocks) and adaptation (against long-term impacts) strategies towards climate change and variability. While some community members have expressed that they do not cope at all or do nothing to cope with either drought or flood, some have adopted several coping strategies such as fishing or selling livestock (Fig 5). Although community members living near and far from the river employ almost the same coping strategies, there are differences in the percentage of pastoralists employing strategies such as fishing, begging and looking for jobs. Fishing was the main coping strategy mentioned by pastoralists living near the river, with half of the pastoralists using it to cope with drought; however, only 7% use it as a coping strategy in the villages far from the river, where begging was the main strategy used. A higher proportion of pastoralists from the villages far from the river look for jobs (43%) and sell their livestock (43%) during drought, compared with those from close to the river (23% and 33% respectively). More people depend solely on relief and government social funds in villages far from the river compared to those living near the river. Villages far from the river do not use replanting and buying fodder as a coping strategy, but villages near the river use these strategies. Only five adaptation strategies were recorded in the study area; relocating livestock to better grazing areas, using drought-resistant crops and livestock, planting early and relocating to other villages. These were mostly used by pastoralists near the river compared to those living far from the river. The main adaptation strategy was changing to drought-resistant crops or vegetables during drought (Fig 5).

Most pastoralists have limited adaptive capacity and ineffective local coping strategies: This was asserted by a few respondents as follow:

*“There is no hope when this happens*, *and life becomes truly difficult*. *The flood washes away the few crops you have*, *and drought brings hunger*. *We sell some of our chickens sometimes in order to buy maize meal*” *H16*

*“How can I survive when I have nothing*, *my livestock are gone*, *and my garden is washed away*? *So I beg from our neighbours and ask family members to help us*” *H12*

“*We reduce the number of meals to save food*, *and since I lost my garden I did not receive any relief from the government*, *this is why you see me carrying these fish I have caught to go exchange it with my neighbours for maize flour to feed my children*” *H2*

*“We do not survive well at all*, *we risk our lives and go to the river to catch fish to feed our children and to sell too*” *H11*

*“Since I have no cattle*, *I just go and beg*, *I have no livestock*. *I try to go fishing to buy food and buy fuel for my generator to work in my garden which is now washed away*” *H10*

### Adaptive capacity and barriers to adaptation

Our results suggest that the adaptive capacity of the Himba community is very low. They currently do not have safety nets, and many have already lost their source of livelihood. The environment they depend on for themselves and their livestock is no longer able to sustain them. The adaptation and coping strategies used by the households are not without constraints. Respondents reported several limitations to effective adaptation, including lack of education (76%), poverty (80%), lack of inputs and equipment for agricultural practices (67%). Other factors limiting them include large extended families, and the most apparent factor was lack of rain.

With regard to the lack of education, one respondent expressed the need for education as follows:

*“Educated people are trying to come up with some solutions*, *and when educated people talk to us about these things*, *many times*, *we think they are against us*, *but they are not*. *In actual fact*, *they are right*. *We sometimes do things that harm the environment*. *We need schools to be taught*, *even old people to be taught*, *though I will probably not go because if I start going to school*, *my family will go hungry as I need to be out there looking for food*. *Who will deal with drought while I am in school*? *So I spend most of my time looking for food*” *H24*

Pastoralists revealed that though they are ready to act and willing to work hard, they are just hopeless due to these constraints. This is clearly expressed by a middle-aged pastoralist who stated that:

*We do not have the capacity to do this ourselves*. *If we had enough water and tools*, *we would have a good livelihood*. *There is hunger in this community*, *and people do not have the means to change anything*, *they cannot buy pumps or tools*, *and there is no rain*, *so we are as hopeless as can be” H41*. *“We need an irrigation system and tools to help us successfully make gardens and make a living*. *We are very poor*. *There is no rain and plants just dry up and die*. *Another problem is*, *people do not have work*, *we look for work*, *but there is no work to make a living*, *so there is no means of livelihood*. *The only solution is a water supply to make gardens*, *and this we currently do not have*. *The government can help us set up irrigations and water supply and help with the fence as well*.

**Fig 6:**
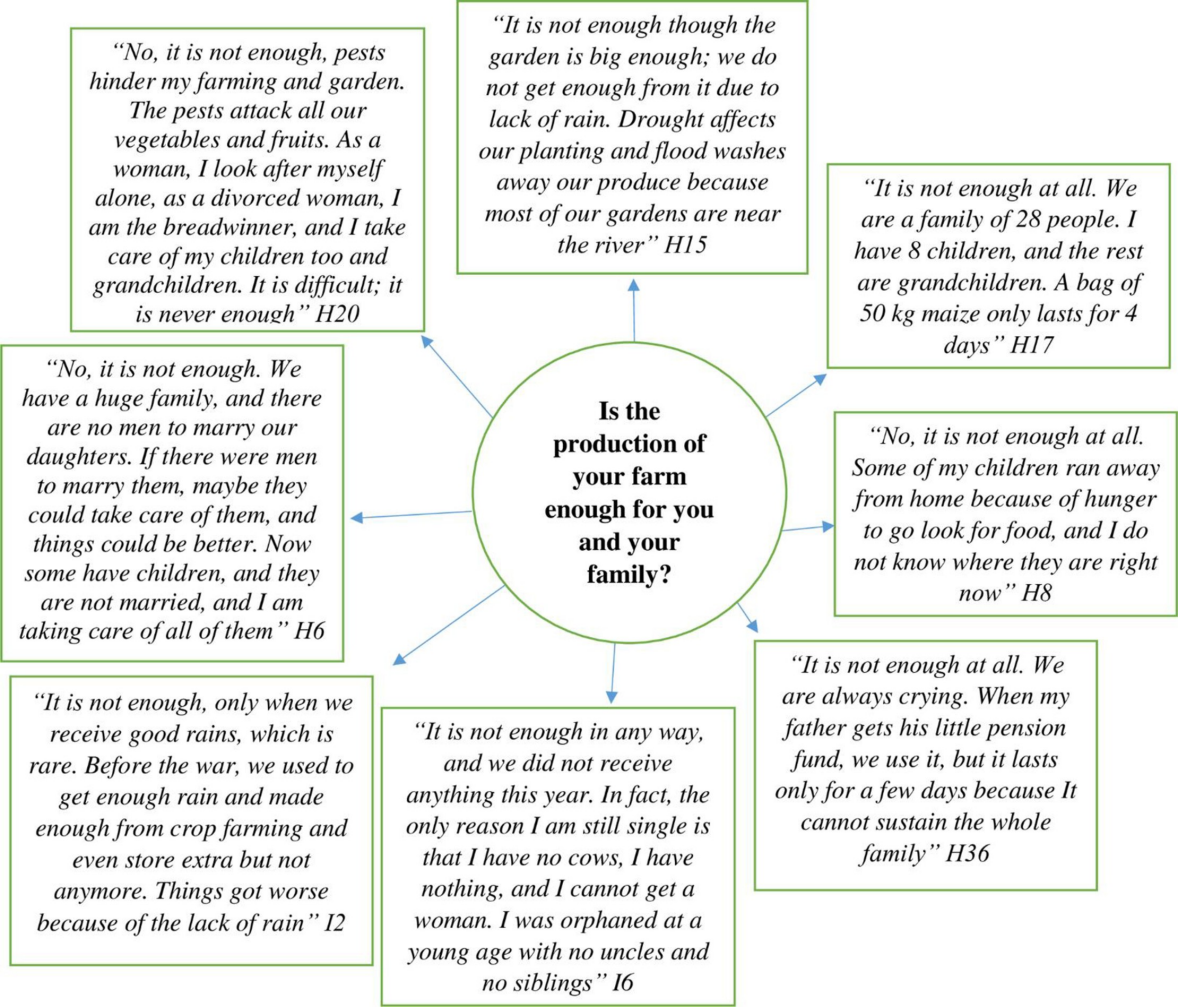
Responses on whether the production of Himba pastoralists’ farms was adequate to support their families.

The majority of the respondents (81%) expressed that the production of their farm was not enough to support their families. The main concern was the lack of rain that had affected their crop production. As a result, many are poor and have no adaptive capacity to cope with climate change or variability. Fig 6 illustrates some of the voices of the pastoralists on their farm production.

### Who is more vulnerable to climate change and variability?

The majority of the respondents indicated that the most vulnerable people to drought and other climate-related disasters are the poor (91%), the widows/widowers (86%), the orphans (46%) and the disabled (20%). One of the respondents expressed that:

*“The widows and widowers and the poorest people are more vulnerable*. *The poor do not have anything*, *and they are hopeless*. *Though the rich are also affected*, *it is not in the same degree*. *The widows are a sad case in this community*” *I4*

One headman also emphasised the vulnerability of the widows in his community:

*“The worst are those who have nothing at all–the poor*, *and there is no word to describe the situation of the widows*, *they are at the mercy of life*. *They have no way to support their children*, *and sometimes they come here asking for food*. *If you give them maize flour*, *they eat just porridge*, *no sauce or anything else to eat with*. *We are left behind in this area*. *The widows are suffering*.”*I5*

**Box 1. Case of a vulnerable household**

Female respondent I9 is a 42-year-old widow, who heads a household of 23 other people; her eight children and grandchildren as well as her mother. Life was better when her husband was alive as he had many cattle and did occasional jobs that brought in some cash. Shortly after he died, they also lost all their livestock, leaving them with no form of livelihood and utterly hopeless. They are currently using someone’s piece of land (0.25 hectare) to grow vegetables, but the flood washed it all away. They survive with the help of government drought relief that gives them bags of maize flour and cooking oil on specific months. This is not enough to support a family of 23, and so they occasionally beg from their neighbours. She concurred that drought had brought disasters:

*“No one works in my family; we mostly depend on drought relief since my husband passed away*. *I am getting tired because it is getting worse*. *In my family*, *a bag of 50 kg maize flour is consumed in 7 days only*” *I9*

They sit under a shade day after day, hoping things will get better. The only shelter they have is a little hut, and most of them sleep outside, most of her children have had malaria, and some have had cholera. Despite this, she still dreams of a better life.

“*The government could help provide us with water pumps and steel to make gardens and upgrade our standard of living*. *We could also be given food for our children and most importantly*, *for my house to be upgraded*. *I want a modern house with running water*, *built with bricks and looking like a lodge*, *so my children do not have to fetch water from the river where there are crocodiles*” *I9*

### Proposed adaptation strategies or suggestions from pastoralists

While some pastoralists suggested that they need their children to be helped with food and clothing, the majority of pastoralists indicated a need for a water supply to make gardens and grow crops, vegetables and fruits so they can be self-sufficient. A piped water supply for gardening would help their irrigation needs, and they would no longer be dependent on the rain. About 87% of the respondents proposed this option. This may be easier for those living near the river, but it is more challenging for those living far from the river, where boreholes will be required. Some respondents also expressed interest in establishing water storage facilities, fodder, feeding programs, and to be provided with drought relief. Table 6 illustrates some of the requests made by pastoralists to help them enhance their adaptive capacity.

**Table 6:** Suggestions by pastoralists on how they can be helped to cope better with drought.

Requested items	Comments
Solar panels, water storage facility	“The government could perhaps give us solar panels to run generators, instead of electricity and fuel. Also perhaps irrigation and water storage facilities. If we are provided with these, it will help us. We also need a water tank with enough pressure like the ones in Etunda irrigation project. They should be erected at a good height to have enough pressure. If we are using energy from the sun, we will not run out” I15
Community garden	“If we all come together and discuss with the government and perhaps an area can be allocated to be used as a community garden where every family has a plot and people will work together. This will help us” H23
	"We want a community garden to help us" H36
	We need to look for a place that can be used by all community members as a garden, and everyone can be given a plot and people can work together" H48
	"Perhaps we can be given a community garden that can support everyone, and each family can be given a plot to grow food. We also need hospitals and cellphone receptions. Water can be provided through digging boreholes" H38
Boreholes	“We just need a water supply, by digging boreholes and providing us with pipes to make gardens. This way, we will not depend on the rain and the government. The soil here is very fertile, and gardens will flourish” I5
	“We have good underground water, and one does not need to dig that deep to get water, so the potential to make gardens is huge, but some people are not able to make gardens because they are not strong enough and our people lack knowledge of gardening and growing crops because we are mainly livestock pastoralists” H24
Capacity building	" We could also be taught and empowered to do business" H30
	"We do not have power and no assistance with even loans to help us with farming activities. Interventions such as small business loans to even start a take away will be helpful to make a living, and because you sign a contract, you need to find a way to be paying it back. These things are not implemented in this community" I5
	“We don’t have the capacity because we are left vulnerable, we have lost all we had. We need money from the government and social funds to help us.” H48
Irrigation systems and gardening tools	"We need an irrigation system and tools to help us to make gardens and make a living successfully. We are very poor. The only solution is a water supply to make gardens, and this we currently do not have. The government can help us set up irrigations and water supply and help with the fence as well. We do not have the capacity to do this ourselves. If we had enough water and tools, we would have a good livelihood. There is hunger in this community, and people don’t have the means to change anything, they cannot buy pumps or tools, and there is no rain, so we are as hopeless as can be" H41
Water supply pipes, water pumps etc.	“Since we have a river, this is our riches, so perhaps we can be assisted with installing pipes to get water from the river and establish gardens far from flood-prone areas, this will help us a lot” I3
	“It will be nice if the government can give me a water pump to make a garden to feed my family, then my heart will be content and more certain of the future. Even as I sit here, I just think I may die. I am always thinking about where will we get food for the next day? The generator will release the stress off me” I11
	“We just need water pumps to become self-sufficient. Moreover, perhaps we can even share in the community and help each other. If one can give me a tap or a water pump, I will not even rest; I will work day and night to feed my family. This will change our lives. We also need ploughing machines as the soil is hard and rocky” H60
	We need pipes and water pumps to make gardens. This village has not received any help at all, unlike other villages. We have no school, no cellphone network, no clinic, no water and no electricity. We travel to other villages for these services, and so this community is left behind in everything" H46
	“We need water pumps to have a water supply for our gardens because it is hard to water by hand and to fetch water from the river. A constant supply of water all year round to grow crops and vegetables all year round is what we need. We also need pipes and seeds” I2
	“If I were the government, I would make sure that people have a water supply to make gardens and fields. If they can install pipes running from the river, then we will not need to depend on the rain anymore” H5
Fishing nets	“We also need nets for fishing in the river; this will help us with food and for selling too" I1

### Ecosystem benefits and threats

**Fig 7:**
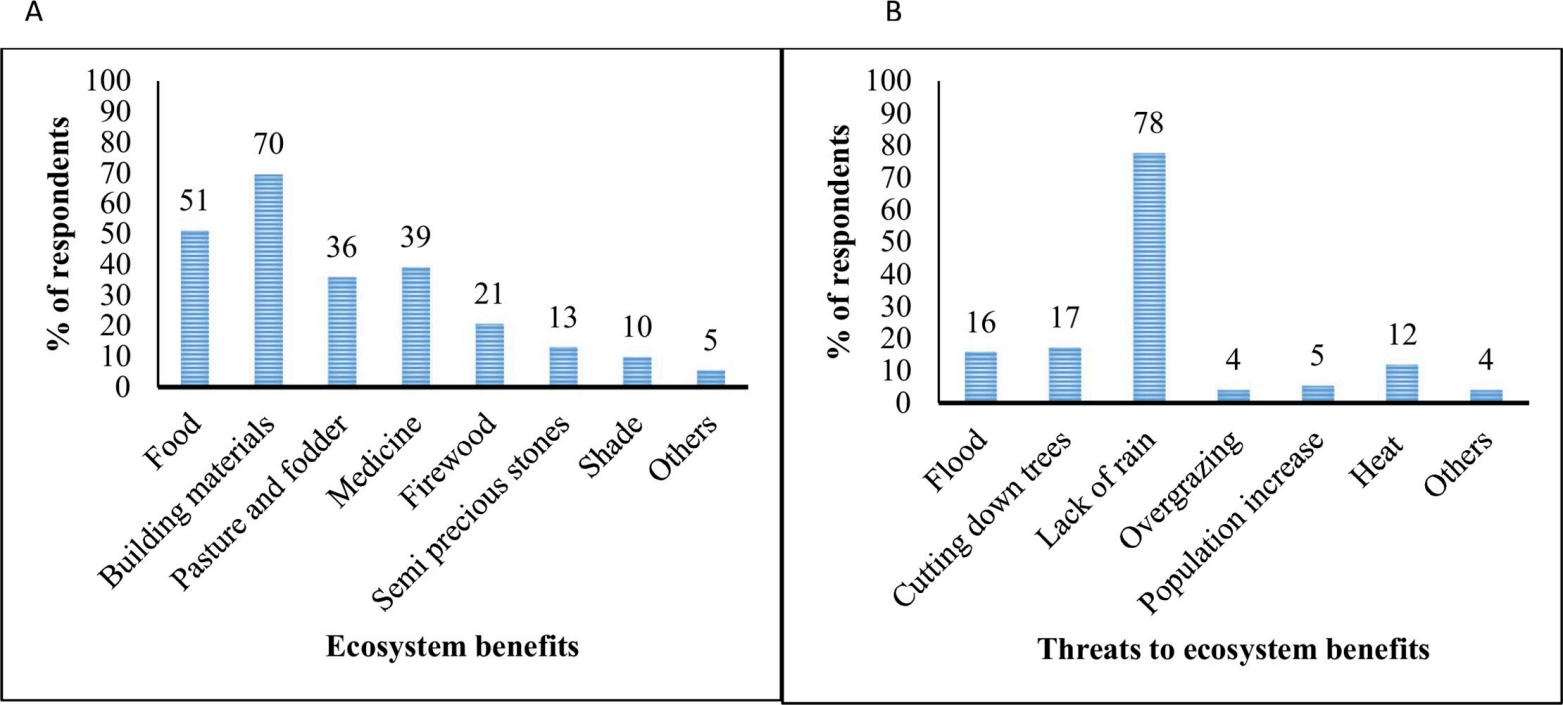
Perception of ecosystem benefits (A) and threats (B) (n = 76, household and in-depth interviews).

The pastoralists in the study area depend mainly on the environment for their livelihood, and rainfall determines their survival. All the respondents concurred that they get benefits from the environment such as building materials (70%), food (51%), medicine (39%), fodder/pasture (36%), and others such as firewood, semi-precious stones and shade (Fig 4A). These benefits are, however, under threat, and many respondents (95%) expressed that these benefits have decreased in availability. The main threat to the ecosystem benefits was the lack of rain (78%) (Fig 7B).

As a result of a reduction in benefits, such as wild fruits, the livelihood of the pastoralists is affected. One pastoralist narrated that:

*“The only benefit we get now is building materials*. *A long time ago*, *we got wild fruits*, *and now there is nothing*. *There is no rain and therefore no fruits*. *We lost a means of livelihood*. *When I was a young herder*, *I survived in the wild*, *I did not have to eat at home*, *there was a lot of food in the wild*, *but this is far from the truth now*” *I11*

Similarly, participants often admitted that in previous times they used to see abundant wildlife in their communities, but only a few remain. This was explained as follows:

*Our wild is very beautiful*, *and when it rains*, *we get fruits*, *honey etc*. *A long time ago*, *people survived on these*; *wild fruits and wildlife*. *Now there is nothing*. *We rarely get fruits from the wild*, *and all the wild animals are no longer here*. *We used to benefit a lot from wildlife*, *and we made different products from these such as clothes made from animal skins*. *We also got fodder and grass for our livestock in the past*. *This area is supposed to be fertile compared to other northern regions and has a rich biodiversity*, *minerals and precious stones*, *and if the government helps*, *there are so many opportunities to make a living*, *but people do not have the assistance*, *no investment*, *no capital*, *no jobs*. *If the government was to provide the machinery*, *perhaps I can employ some people” I5*

Pastoralists have also experienced local extinction of some plants and animals. There are wild food species, mostly edible which have become locally extinct or are in short supply since the past years. Some elderly members of the community referred to the lost benefits as “the good old days”:

*“Some of the benefits are no longer here; we do not see things like mopane worms anymore*. *When we were growing up*, *we had these in abundance*. *They are nowhere to be found in this area now*. *The good old days are gone” H17*

*“Some of the benefits are no longer here*, *we do not see things like pangolins anymore or mopane worms*, *when we were growing up*, *we had these in abundance*. *The pangolins are totally gone*, *no longer in this community” H41*

**Fig 8:**
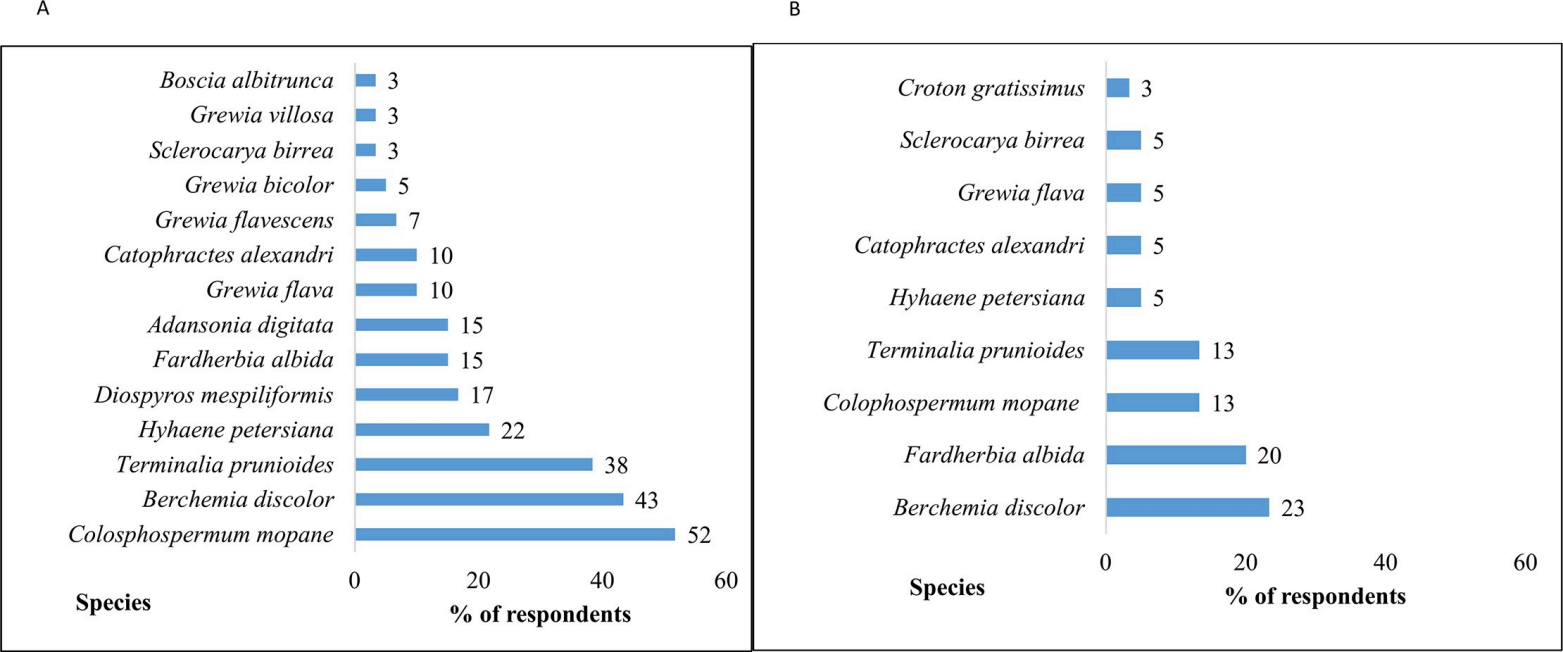
The most important (A) and most threatened (B) species to the pastoralists in the study area (n = 76, household and in-depth interviews).

Trees are the most important plant types to the pastoralists as they get more benefits from them, especially considering that grass only grows when it rains. *Colophospermum mopane* was ranked as the most important species by the majority of the respondents (52%) followed by *Berchemia discolor* (43%) and *Terminalia prunioides* (38%). The most threatened species was *Berchemia discolor* (23%) followed by *Faidherbia albida* (20%) (Fig 8).

### Biodiversity interventions to protect ecosystems

We found that there are currently no biodiversity interventions at the community level to tackle the problem of drought or flood in the area or protect the environment. However, the government has put up some measures such as restricting people from cutting down trees as explained below by one of the key informants:

*“There is government intervention to limit cutting down trees unnecessarily*. *Perhaps the government can help define boundaries*, *so everyone is given their land*, *and the land can then be divided*, *and people will have a sense of ownership perhaps that will encourage people to take care of their land” K3*

Not many people follow these restrictions and so deforestation and overgrazing are still on the increase. The following excerpt taken from two interviews sheds more light on community involvement to counter biophysical impacts of climate change in the area.

**Interviewer**: What type of practices are there in order to protect natural resources?

**Respondent H41**: Nothing, perhaps the government can help us fence off the land…

**Respondent I15:** Nothing much really. Some areas are really overgrazed, and some people are forbidden to graze in some areas by some selfish people, leading to more overgrazing as they continue to graze in one place only.

Some pastoralists indicated that there are environmental problems in the area such as overgrazing and deforestation and social problems such as unemployment has exacerbated their situation. One respondent shed light on some environmental problems:

*“Yes*, *we have overgrazing and deforestation in the area. Many people keep constructing houses, and for this, they need building materials from trees. We keep animals because that is our livelihood, we are not educated, we do not have jobs, and so this is how we survive, through livestock rearing” I3*

## Discussion

The objective of this study was to investigate pastoralists’ perceptions, experiences and understanding of climate change and its associated impacts on their livelihood in Epupa, Kunene Region Namibia. Our results suggest that the pastoralists in the study area have experienced and perceived climate change and variability, but they lack scientific knowledge and understanding of what climate change is. Their understanding is limited to what they have experienced, in their daily lives such as changes in rainfall patterns and not necessarily on what they have learned, for example, from school. This is explained by lack of education as most of the pastoralists have never attended school. Several studies [57] have found similar results in Africa. Although scientists have convincingly established causes of climate change, how people perceive the issue may be solely through the experience of damages [58], more so for people living in remote areas with no access to climate information and education.

The majority indicated that climate change is not important to them. This is cause for concern because people can only value or act on what they know and what they understand. If people do not have climate change knowledge or information, how can they ever be climate change-ready? Climate information is one of the crucial factors for effective adaptation to climate variability in pastoralism [3,8]. Some pastoralists indicated that climate change is caused by God, and it is, therefore beyond human perception and elucidation. Several studies in Africa have found similar results [10,17]. For instance, in Kenya, local communities regarded droughts as acts of God beyond human control [17]. Beliefs concerning climate change determine the perceived seriousness of climate change [59]. If people do not see a relationship between what they perceive and what affects them in their local environments and climate, they may regard them as two separate issues [60]. Hopkins [61] emphasised that climate can be understood through formal meteorological statistics or as a supposition formulated by local perceptions, actions, and cultures. There is, therefore, a difference in perception derived from personal experience and statistical description and science. Some studies [61,62] have established that knowledge about climate change science significantly affects opinions about climate change. Broad understanding and information-sharing can lead to significant public opinion cultivation [62]. Spear and Chappel [35] stated that limited information on adaptation is a barrier to the adoption of new agricultural interventions in Namibia and local standards, customs and beliefs influence adaptations to climate change.

Though climate change is a notion pastoralists do not understand; our findings also indicate that they have perceived climate change or variability. Hopkins [61] highlighted that while the term ‘‘climate change” is highly recognised by the general non-scientific public, understandings of its manifestations are different, contrasting, and complex. The main climate changes perceived by pastoralists in Kunene include limited rainfall, recurrent droughts and floods. Other studies in several parts of Africa [35,63–65] also found similar results. In pastoral production systems, climate change and variability may manifest as flood and drought events [66]. Some of the drought events mentioned by the pastoralists correspond with the existing meteorological-based empirical evidence of recorded data such as the drought of 1992, 2007 and 2011/2012. Analysis of meteorological data for the 50 years (1962 to 2019) shows variability and a general decline in rainfall in Kunene. The perception that the rainfall has declined also correspond with the observed pattern with rainfall data. Previous studies [37,38,67] in the study area corroborate cattle owners’ experiences of severe drought. Similar results of pastoralist perception of declines in rainfall have been reported in many countries across Africa [63,68–70].

### Traditional weather forecasting and indigenous knowledge

Our study revealed the use of indigenous knowledge by some elderly pastoralists, such as slaughtering a goat and examining its ribs and gut glands—which were deemed useful for predicting the rain. However, the poor will not want to slaughter a goat from the few they have. Other respondents indicated the use of biological indicators such as certain plants and animals to predict the rain. Many studies in Africa have documented indigenous knowledge used by pastoralists and farmers in predicting weather and seasonal events [8,33,34,65]. Some studies [8,11,65] have shown that the use of weather forecasts positively influenced adaptation to climate variability. So, the use of local knowledge to predict rain is still very useful to many in the community. However, many studies [32,35,63,71] have shown that traditional forecasting has become unreliable because of increasing climatic uncertainties and presented new challenges.

### Impacts of drought on the livelihood of the pastoralists in Kunene

Kunene region has had a decade of persistent drought, and many pastoralists lost their main form of livelihood–their livestock. Many resorted to begging and depended on drought relief food for survival because their granaries were empty [72]. With a lack of opportunities and weak adaptive capacity, the drought has handicapped the community in terms of access to food. The danger of this to society is that, instead of people becoming useful citizens and concentrating on developing their communities and education, people spend all their energy and time looking for food. The poor invest their time trying to survive instead of progressing themselves, which stresses that poverty indeed is a trap. Due to losses of their livestock, many have fallen into poverty. Hallegatte and others [73] emphasised that poverty is not fixed, and poverty reduction is not a uniform, one-way process. Eventually, some people may be able to get out of poverty, while others may be dragged into poverty [73]. In this study, respondents expressed that the poor, widows and orphans were more vulnerable to the impacts of climate change. According to Opondo and others [74], people who are poor and marginalised generally have the least safeguard to face even modest climate threats. It is, therefore, easy for them to fall into poverty traps. Even those who are not poor are still at risk of being dragged into poverty due to climate-related disasters depending on their asset portfolios. For example, rich pastoralists could be dragged into poverty if they lose all their livestock in a drought. This is precisely the case in the present study. Pastoralists who had over 50 cattle, who were able to produce milk for their families and make an income from their livestock, were left hopeless after the drought. Some have tried to recover, but recurrent drought pushed them further into poverty. Many pastoralists’ assets, such as livestock and gardens, offer little security because they are sensitive to climate change [27]. Caney [75] is of the opinion that poverty should be addressed in light of climate change because it is a significant driver of global poverty. Due to drought, pastoralists are faced with food insecurity and hunger. Some 80% of respondents indicated that, because of drought, the production of their farms is not enough to support their livelihood. The ongoing uncertainty about rainfall, feed and water scarcity makes their situation dire.

### Coping strategies, preparedness and barriers

In this study, preparedness in the face of climate change was weak as many respondents concurred that because drought or flood events catch them off-guard, they do not prepare for either drought or flood. Some people who use local knowledge still do not prepare because they do not have the capacity to do so. However, they still find a means to cope with climate-related disasters. Some coping strategies include fishing, begging, or looking for occasional work. These strategies come with their own challenges, such as lack of job opportunities or the danger of fishing because of crocodiles. Hence, these coping strategies are not really effective and leave most pastoralists still vulnerable. Different environments offer different opportunities to the pastoralists. For example, those living near the river have more coping options. They have increased access to water and can resort to fishing during drought, despite the danger of crocodiles. Living close to the river also provides access to tourists who may buy crafts and other products, and access to occasional work.

Conversely, those living far from the river have to travel long distances to go fishing, and as a result, only a few people do so. On the other hand, coping strategies such as selling semi-precious stones and collecting wild vegetables and plant parts are used by people living far from the river as they are closer to the mountains where these items are found. Villages far from the river do not use replanting and buying fodder as a coping strategy, probably due to low water availability and lower access to shops and transport links to urban centres with larger markets. The poorest households depend more on forest products for their coping and adaptation options because harvesting usually involves limited financial, physical, or human capital [27]. Although some pastoralists mentioned relief food as a coping strategy, they also indicated that the relief was unreliable and ineffective, often not enough and only provided for a few months in a year. Reliance on relief food could limit adaptation in the sense that it can lead to dependency unless the assistance also enhances their ability to produce their own food [22]. According to Birch & Grahn [7], many developing countries do not invest in protecting livelihoods in drought-prone areas but have instead relied on food aid. This has contributed to a depletion of pastoralists’ resource base; reducing their adaptive capacity. Investing in opportunities for income generation that are complementary to pastoral production and that promote alternative livelihoods is more critical and can be done if it is prioritised.

For many pastoralists, lack of education is a barrier to adaptation in the study area, and this corroborates with several pastoral communities across Africa, such as in Somalia [76] and Burkina Faso [77]. Investment in education to improve literacy levels is, therefore, crucial in addressing cyclic drought vulnerability [78]. Pastoralists’ drought response mechanisms are disparaged by increasing land degradation, limited access to information, lack of education, skills and access to financial services [76].

### Adaptive capacity and proposed adaptation options

At the moment, the adaptive capacity of the pastoralists in Kunene is low, and they cannot improve their livelihood on their own. The loss of livestock has pushed the Himba people to consider alternative sources of livelihood. The majority seem to have realised that making gardens to diversify their livelihood is the way forward, and thus a majority of them requested water pumps or boreholes and pipes as a way to increase their adaptive capacity. Many believe that this will give them a perpetual solution amidst drought. Livelihood diversification among African pastoral communities is not a new phenomenon. Pastoral peoples in Africa, and many areas around the world, have been diversifying their economies since time millennial [79]. There are many records of where this option has worked, such as the Maasai in northern Tanzania [79,80], Kenya [81] and Ethiopia [82,83]. Ayeri [17], suggested that the adaptive capacity of pastoralists can be improved if national policies promote coping or adaptation approaches that are already being employed by pastoralists in their communities. This may be challenging for governments of most developing countries such as Namibia who are already struggling financially. There are still various ways to help pastoral communities, for example, raising money through several NGOs or through international organisations that support community projects with the help of researchers. This has been done successfully before [84], especially when using a participatory strategy.

### Biophysical impacts, ecosystem benefits and opportunities for EbA

An upsurge in extreme rainfall patterns, especially in Africa, causes drought and water stress, affecting ecosystems that many depend on [74]. All the respondents expressed that they have lost livelihood benefits from the environment because of lack of rain, making their situation even more severe. Ecosystem benefits such as wild fruits and other forest products which supplemented their pastoral livelihood are no longer available, and some plant species are locally extinct. Despite the reported biophysical impacts such as loss of biodiversity, drought impacts on livestock and vegetation, and reduction of ecosystem benefits affecting livelihoods, there are currently no biodiversity interventions at the community level. The pastoralists have, however, realised their need to do something to save their livelihood. The most common suggestion is diversifying their livelihoods through growing food, and this will open the door to explore Ecosystem-based Adaptation strategies, specifically ecological restoration. Ecological restoration may become a useful adaptation tool to help vulnerable communities adapt to climate change. However, restoration needs to be considered within the whole adaptation strategy, because an EbA strategy cannot be a stand-alone activity; there are social, governance and environmental factors that also need to be considered and incorporated within the adaptation strategy. Now that local knowledge is established, and pastoralists have voiced their perception of climate change, benefits and what is important to them, implementing EbA will take into consideration the needs of the communities and their suggestions, to explore options that will enhance resilience and subsequently improve their livelihoods. One way to introduce EbA in places like Kunene while still addressing the pastoralists need–growing food, is to include revegetation, tree planting campaigns, implementing exclosures, among other activities. This could be done through a participatory approach where both researchers and communities bring something to the table. Often, the challenge is finding a way for scientists and local communities to work together, but if that bridge is built, then it becomes easier to move forward. Pastoralists are willing to work to become self-sufficient, and they have indicated their adaptation need–water supply. Putting their considerations into action requires careful consideration of how this can successfully be implemented. According to McLean [32], the incorporation of both traditional wisdom and the scientific methods to adapt to climate change impacts provides a pathway to new partnerships and inventive ways of thinking. The engagement and full participation of community members will make a big difference. The opportunity for the pastoralists to grow food will offer a diversified resource base. Several studies have shown that western technologies and concepts may not always fit the cultural context of many developing nations [85]. Sustainable solutions, therefore, need to draw on indigenous knowledge that is compatible with the local culture [85].

Restoration and conservation strategies falling under EbA approaches can create a platform for change. EbA, in itself, is not a panacea. It builds on and is complementary to other approaches such as Community Based Adaptation or Sustainable Livelihood. EbA is a human-centric approach that intentionally merges conservation and socioeconomic goals to sustain livelihoods and enhance people’s adaptive capacity to climate change [86]. The results of this study will contribute to the formulation of EbA strategies. It provides information on people’s concerns, and this can then feed into the development of effective interventions. As a follow-up activity, the researchers have raised money to meet the need of the pastoralists to grow food, and the leaders in the community have allocated land to be used in this initiative. This has paved the way to incorporate restoration activities, and community members are on board to work together with the researchers. Two other separate studies have also been conducted–exploring restoration techniques and assessing land degradation in the same area–which will also inform the study on EbA strategies.

There is evidence that increasing biodiversity can enhance ecosystem functionality and stability, and hence various ecosystem services [87]. Ecosystems play important roles in livelihood resilience in the face of climate change; acting as safety nets for many vulnerable communities [88]. The idea here is to draw on pastoralists' knowledge to implement management strategies that support resilience, and ecological restoration is one option. Again, it is important to note that ecological restoration could enhance resilience, but not as a stand-alone strategy [28]. It is through initiatives like these that pastoralists may be assisted to improve their livelihood and tackle the impacts of climate change.

## Conclusions

This paper has endeavoured to illustrate pastoralists’ perceptions of climate change and variability and explored the adaptation strategies of pastoralists in Epupa constituency, Kunene region, Namibia as well as impacts. Our study revealed that pastoralists living in Epupa constituency in Kunene have little or no adaptive capacity in the face of climate change. We found an overall lack of scientific understanding of climate change in this pastoral community, and this is cause for concern considering the seriousness and the threat climate change presents. Based on this study, we recommend instituting climate change awareness campaigns at the community level to disseminate climate change information. There is also a need for early warning systems to help pastoralists in their preparedness. Developing climate information and early warning systems in pastoral communities is a crucial component for building pastoralist resilience [63] but is currently lacking in the Kunene Region.

The droughts are recurrent and severe, and the pastoralists have no safety nets. There are many other cases like this all over the world, especially in the Third World, where people are deprived of their livelihood and their futures threatened. Nothing much has been done to address this serious concern in Kunene, and the situation is getting worse. Current coping and adaptation strategies employed by the community are not feasible in creating sufficient income, nor are they sustainable because of the changing climatic conditions and diminishing availability of natural resources. As a result, the community is showing signs of abject poverty. With no interventions, Kunene will continue to experience the effects of persistent drought and erratic rainfall. So, what can be done to change this?

Real solutions are required, and a lot needs to change, not just at a global level but starting at grassroot levels. The climate situation calls for solutions that will make a difference, not just giving people handouts, akin to giving them fish without teaching them how to fish, but working together with the communities as part of the solution, to empower them to become self-sufficient and enhance their adaptive capacity. This should become a global priority. Solutions that will work are initiatives that will empower the people and help them realise and encourage their potential. Projects could ideally involve the people and provide skills-based training along the way. There is a need to provide location-specific and needs-based information to pastoralists to help them make informed decisions that will empower the farming communities so that they evolve suitable coping and adaptation strategies to climate change-related risks and uncertainties. Empowering farmers as active agents of change stimulates action and long- term adaptability [28]. We recommend EbA strategies to be established in communities like these as a way to increase the resilience and adaptive capacity of the vulnerable communities. This could potentially focus on ecological restoration, which is increasingly acknowledged as an adaptation tool to climate change.

## Supporting information

S1 Appendix Household interview questions. (PDF)

S2 Appendix In-depth interview questions. (PDF)

S3 Appendix Key informant interview questions. (PDF)
