# Supplementary material for: No safety net in the face of climate change: The case of pastoralists in Kunene Region, Namibia
Source: PLoS One. 2020 Sep 15;15(9):e0238982. doi: 10.1371/journal.pone.0238982 (PMC7491742; doi:10.1371/journal.pone.0238982)
Supplement: S1 Appendix — (PDF) [file pone.0238982.s001.pdf]

Climate Change vulnerability and adaptation assessment

Household surveys

Background

Gender of household head and marital status:

i) How many people live in this household?

Age	Gender	Occupation

ii) Main form of livelihood

……
……

General Climate Change knowledge and awareness

1. Have you heard of climate change? Yes ☐ No ☐
2. What do you know about it?

……
……
……
………………………………………………………………………………………………

3. How important is the issue of climate change to you personally?(***tick the appropriate box***)

Not important	
Quite important	
Important	
Very important	

4. What do you think **causes** climate change?

……
……
……
………………………………………………………………………………

5. What **impacts**, if any, do you think climate change may have?

……
……
……
………………………………………………………………………………………

6. Do you think anything can be done to tackle climate change?
   Yes ☐ No ☐ Don't know ☐

   If yes, what do you think can be done to tackle climate change?

……
……
…………………………………………………………

7. Who do you think should have the **main** responsibility for tackling climate change?

Please tick one box only:

International organisations (e.g. the UN)	
The national government	
Local government	
Business and industry	
Environmental organizations	
Individuals	

8. Who do you think is mostly affected by climate change/drought in this area and why?

……
……
…………………………………………………………

9. Do you have access to weather and climatic information? Yes or no

………

10. Have there been delays in the start of the rainy season and early ends of the rainy season in the past five years. If yes, why do you think so?

……
……
……
……
……

Natural disasters and existing coping strategies

1. When often do you experience flood and drought?

……
……………………

- When was the last flood or drought event you experienced?

……
……………………

2. How has drought/flood and climate change affected you? Tick appropriately

**Impact**	**Yes/No**
Agricultural production hampered	
Loss of trees/gardens/house	
Loss of livestock or domestic animals	
Loss of income	
Health hazard	
Scarcity of drinking water	
Others	

3. How do you cope with drought or floods? **(coping)**

……
……
……
……
……
……

4. Do you do anything to prepare for flood or drought?(***yes or no)*****(preparedness)**

……
……

- If yes, what do you do?

……
……
……
……
……
……

- How long have you been doing this and has it been effective?

……
……
……
……
……
……

- Where did you learn it from?

……
……

5. Do you get administrative mitigation and relief measures? (***yes or no)***

If yes, name them.

……
……
……
……
……

-Do you think these are effective? Why and why not?

……
……
……
……
……

6. Are there any plant indicators or any other biological indicators of floods or drought? If yes,have these been accurate indicators? Why or why not?

……
……
……
……
……

Agricultural skills and productivity

1. Have you changed the types of crops you grow or types of animals you farm with since you started farming?

Change……
……

Why?...
....

Result……
……….....

2. Who do you discuss agricultural problems with?

……
……

-Do you speak with the agricultural officer?

……
……

3. Have you received agricultural training? Yes------------No----------

Why? ....
………

4. Is the production of your farm enough to cover the food needs of you and your family? ***(yes or no)***

Comment:

………
………
………
………
………

5. From what your family consumes, how much produce is bought and how much produce is produced on the farm? ***(tick appropriate box)***

Everything is bought	
Nothing is bought	
50% bought and 50% produced	
20% bought and 80% produced	
20% produced and 80% bought	
30% bought and 70% produced	
30% produced and 70% bought	
Other:	

INDICATORS OF SENSITIVITY AND CAPACITY OF ADAPTION TO PHYSICAL CAPITAL

Condition of roads and access

1. Does climate change affect your routes of access? (with regard to all family members), if yes how?

………
………
………
………
………

2. What are the roads like from your farm to the different services such as schools and hospitals?

………
………
………
………
………

Quality of accommodation

1. What material is your home built with?

………
………
………
……………………………………………………………………………………………

2. Does climate change affect the structure of your accommodation? ***(if yes, prompt for possible ways in which they are affected)***

……
……
……
……
……

Access and availability of water

1. Do you have water, electricity and sewerage? (***tick appropriate box)***

None	
Only water	
Only electricity	
We have water and latrines	
We have water, electricity and latrines	
We have water, electricity, sanitation and sewage	

2. Has climate change affected your Access to or the availability of water? (with regard to all family members) if yes, how?

……
……
……
……
……

3. Where does the water come from for your consumption and/or agriculture?

……
………………………………

4. How far do you have to travel to obtain water?

……
………………………………

5. Do you have water all year round? Yes__ No__

6. Is the water for your consumption drinkable? Yes__ No___ if not, how do you make it drinkable?

……
……
………………………………………………………………

7. Have there been any cases of Cholera?

……
……
………………………………………………………………

Ecosystem services and conservation

1. What benefits do you get from the environment? (Prompt on different types of services, provisioning etc)

Benefit	Decreased	Increased	No longer available	Importance (Not imp, Impor, Very impor)

2. What plant species are important to you and why? Did their population increase or decrease in the past five years?

**Species name**	**Uses**	**Decreased**	**Increased**

3. What are the main threats to these species and to various ecosystems at large?

……
……
……
……
……

4. Which plants do you think are mostly affected by climate change in this area?

……
……
……
……
……

5. Does climate change adversely affect natural resources? Yes______ No_______
   How?

……
……
……
……
……

6. What type of practices are there in order to protect natural resources?

……
……
……
……
………

List all the land related problems in this region

1.

2.

3.

4.

5.

6.

What causes these problems?

……
……
……..
