# Supplementary material for: No safety net in the face of climate change: The case of pastoralists in Kunene Region, Namibia
Source: PLoS One. 2020 Sep 15;15(9):e0238982. doi: 10.1371/journal.pone.0238982 (PMC7491742; doi:10.1371/journal.pone.0238982)
Supplement: S2 Appendix — (PDF) [file pone.0238982.s002.pdf]

Climate change adaptation and vulnerability

In-depth interviews

Name of village and location (GPS):

Farmer's characteristic

Age: ………

Gender: ………

Farming generation: ………

Farm size or livestock number: ………………………………………………………………………………………..

How long have you been farming? …………………………………………………………………………………….

Market activity: ………

What was your background before farming?(Education level, Employment, Scientific background)…….

Do you identify as a conventional, sustainable or organic farmer?
………

Is the production of your farm enough to cover the food needs of you and your family? ***(yes or no)***

Comment:……
………
………
………
……….

Do you depend on farming alone or is there anything else you do for livelihood?

………
………
………
………
………
………

Climate-related Information and impacts:

1. Tell us what you know about climate change?

………
………
………
………
………

2. In general, where do you get information about weather and climate?

………
………

3. How have changes in climate affected your farm since you began farming? (Temperature, precipitation, patterns)

………
………
………
………
………
………
………
………
………

4. What specific management practices on your farm are directed at dealing with climate?

………
………
………
………
………
………
………

5. What could help you to better prepare for climate variability?

………
………
………
………
………
………
………

6. What are the main risks you are concerned with as a farmer (rank)?

………
………
………
………
………
………

7. Apart from climate change, what are some factors affecting you as a farmer?

………
………
………
………

……
……

8. Do you feel prepared to face long-term changes in climate as caused by global change?

……
……
……
……
……
……
……
……
……
……

Natural disasters

1. Explain how drought has affected you and your family for the past 20 years in terms of livestock loss, family income and other areas impacted by drought or flood

……
……
……
……
……
……

……
……
……
……
……
……

2. How many livestock do you currently own and how many did you lose due to drought?

……
……..

3. How you prepare for the drought? Does it work? What will you do differently in the future in response to drought?

……
……
……
……
……
……

Ecosystem services

1. What benefits do you get from the environment?

....
....
....
....
....
....

2. Which benefits are more important to you and why?

....
....
....
....
....
....

3. Has there been a decrease in these benefits over the years?

....
....
....
....
....
....

4. How has this affected you? To what extend has this affected you?

....
....
....
....
....
....

5. Identify threat for each benefit you mentioned

....
....
....
....
....
....

6. Who do you think is mostly affected by these disasters in your community?

....
....
....
....

………
………

7. What do you think needs to be done to help these people to withstand climate change impacts?

………
………
………
………
………
………

Overgrazing and land degradation

1. Do you think there is enough grazing land?

Explain……
………
………
………
………
………………………………

2. Is there any land related problem such as overgrazing, deforestation etc in the area?

-What do you think causes these?
………
………
………
………
………
………………………

-Has these problems increased or decreased over the past 5 to 10 years? Explain

………
………
………
………
………
………

3. Is the community doing anything to tackle these problems? Explain.

………
………
………
………
………
………

……
……

4. Has there been intervention from the government to tackle these peoblems?

……
……
……
……
……
………………………
