# Supplementary material for: No safety net in the face of climate change: The case of pastoralists in Kunene Region, Namibia
Source: PLoS One. 2020 Sep 15;15(9):e0238982. doi: 10.1371/journal.pone.0238982 (PMC7491742; doi:10.1371/journal.pone.0238982)
Supplement: S3 Appendix — (PDF) [file pone.0238982.s003.pdf]

Climate change adaptation and vulnerability

Key informants interview

Background

Gender:

Age:

Occupation:

General Climate Change Knowledge

1. What do you know about climate change?

……
……
……
……
…………………………………………………………………………………………………

2. How has climate change impacted people in this area?

……
……
……
……
…………………………………………………………………………………………………

3. In your opinion, what is the most important problem or issue around climate change that should be addressed at the present this community?

……
……
……
……
…………………………………………………………………………………………………

4. In your opinion who is more vulnerable to climate change in this area and why?

……
……
……
……
…………………………………………………………………………………………………

5. Has outreach to the most vulnerable populations on the impacts of climate occurred (or has the idea to discuss it been initiated) in this community?

……
……
……

………
……

6. How has drought and flood affected the people in this community?

………
………
………
………
……

7. Are there any reparations or initiatives to help those affected by flood and drought?

………
………
………
………
……

8. To the best of your knowledge, are climate change awareness campaigns carried out in rural communities?

………
………
………
………
……

9. To the best of your knowledge, do the schools in which your office operates teach students about climate change?

………
………
………
………
……

10. The following table lists a number of potential adaptation measures. Please indicate (x) which of these are planned or have been implemented in your region as a response to climate change concerns, and which of these you deem necessary and/or effective in addressing climate change-related problems. Please add additional measures if necessary.

**Adaptation measure**	**Implemented**	**Planned**	**Effective/necessary (but not planned yet)**	**Not relevant/ necessary**
Technical flood protection (e.g. raises dykes, enlarge reservoirs, upgrade drainage systems.)				
Livelihood diversification				
Natural retention of flood water (e.g. floodplain restoration, change of land use)				

Adaptation measure	Implemented	Planned	Effective/necessary (but not planned yet)	Not relevant/ necessary
Restriction of settlement/building development in risk areas				
Improving forecasting and information				
Improving insurance schemes against flood or drought damage				
Restriction of water uses				
Landscape planning measures to improve water balance (e.g. change of land use, reforestation, reduced sealing of areas)				
Economic incentives and financial mechanisms				
Awareness-raising or information campaigns				
Others, please specify:				

Ecosystem services

1. What are some of the ecosystem services derived from biodiversity in this area? Which ones are more important to the people in this community and why?

………
………
………
………
……

2. Has there been a decrease/increase in the availability of these services? Why do you think so?

………
………
………
………
……

3. Do you see climate change as a threat to the environment and ecosystem services? Explain

………
………
………
………
……

4. Are there policies to manage ecosystem services in this area?

………
………
………

………
……

5. What opportunities are provided such as employment due to the harvesting of ecosystem services?

………
………
………
………
……

6. What adaptation practices are currently in place to reduce the vulnerability of natural resources to climate change impacts?

………
………
………
………
……

Environmental problems

1. What are the main socio-ecological issues in this area and what are the approximate causes?

**Problems**	**Causes**

2. Are there policies put in place to deal with these issues at local and regional level? Explain

………
………
………
………
……
